# Phenotypic variations of naturally grown *Iris persica* L. accessions as revealed using multivariate analytical methods

**DOI:** 10.1371/journal.pone.0354156

**Published:** 2026-07-27

**Authors:** Ali Khadivi, Alireza Khaleghi, Yazgan Tunç

**Affiliations:** 1 Department of Horticultural Sciences, Faculty of Agriculture and Natural Resources, Arak University, Arak, Iran; 2 Republic of Türkiye, Ministry of Agriculture and Forestry, General Directorate of Agricultural Research and Policies, Hatay Olive Research Institute Directorate, Hassa, Hatay, Türkiye; Kerman University of Medical Sciences, IRAN, ISLAMIC REPUBLIC OF

## Abstract

*Iris persica* L. is a high-value ornamental species naturally distributed across montane regions of Iran, yet its intraspecific morphological variation remains underexplored. Understanding phenotypic diversity among wild accessions is critical for identifying superior genotypes suitable for conservation and horticultural breeding. This observational study was conducted on 78 naturally occurring *I.* *persica* accessions collected from seven ecogeographical regions of Markazi Province, Iran. Each accession represented a single independently growing individual plant, and 39 morphological and colorimetric traits were evaluated using multivariate statistical analyses. The results revealed considerable variation among accessions. Descriptive statistics showed that flower diameter ranged from 34.52 to 80.56 mm, fall length from 35.39 to 57.70 mm, and spathe length from 25.86 to 64.56 mm. Principal component analysis identified ten principal components explaining 82.46% of the total variation, with the first component (33.30%) driven primarily by flower size, fall length, and spathe length. The heatmap analysis clustered accessions into four distinct morphological groups, indicating clear phenotypic differentiation. Correlation analysis revealed significant positive associations between flower diameter and flower surface (*r* = 0.87, *p* < 0.01), fall length and flower surface (r = 0.76, p < 0.01), and flower diameter and flower length (*r* = 0.76, *p* < 0.01). Stepwise regression analysis indicated that fall length, flower diameter, and spathe length were key predictors of flower diameter. This study demonstrates extensive morphological diversity in *I. persica*, providing a strong basis for the selection of superior genotypes for ornamental breeding programs. Accessions such as ‘Alibolaghi-6’, ‘Sorkhe-4’, ‘Sorkhe-14’, ‘Shahbaz-11’, ‘Sorkhe-11’, ‘Sorkhe-12’, ‘Sorkhe-6’, ‘Palangdarreh-8’, ‘Sorkhe-5’, and ‘Bolagh-8’ exhibited outstanding floral characteristics and are recommended as potential candidates for future cultivar development and conservation strategies.

## 1. Introduction

The genus *Iris* L. (Iridaceae), encompassing over 300 recognized species, exhibits remarkable diversity in morphology, phenology, ecological amplitude, and ornamental appeal [1]. Distributed across temperate zones of the Northern Hemisphere, particularly in the Mediterranean Basin and southwestern Asia, *Iris* species have long attracted the attention of botanists, horticulturists, and conservationists for their striking floral architecture and ecological plasticity. Among these, *Iris persica* L., native to montane and submontane regions of the Middle East, represents a highly adaptive and visually distinctive species, often regarded as a valuable genetic reservoir for ornamental breeding and ecological restoration programs [1–3].

*I. persica* is characterized by its dwarf stature, early spring flowering, and vibrant floral pigmentation—traits that not only contribute to its ornamental potential but also reflect complex adaptive responses to harsh highland environments [4]. Despite its ecological and horticultural relevance, comprehensive studies on the intraspecific morphological variation and population-level differentiation of *I. persica* remain scarce. The intricate taxonomy of *Iris* species primarily stems from recurring hybridization processes, widespread polyploidy, and pronounced phenotypic plasticity, all of which hinder precise species delimitation and the resolution of intraspecific variation [5]. Genetic diversity in plant species is commonly assessed using both molecular and phenotypic approaches. Molecular marker systems, such as simple sequence repeats (SSR), inter simple sequence repeats (ISSR), amplified fragment length polymorphism (AFLP), and single nucleotide polymorphism (SNP)-based markers, provide direct information on genome-level variation, population structure, gene flow, and genetic relationships among populations [6]. However, in wild ornamental species, especially when molecular resources are limited, morphological characterization remains a practical and informative first-line approach for evaluating diversity, identifying distinct accessions, and selecting promising germplasm for conservation and breeding [7]. When combined with multivariate statistical tools, including factor analysis, cluster analysis, and regression-based modeling, morphological descriptors can effectively reveal patterns of phenotypic differentiation and provide preliminary insights into the underlying genetic variation among natural populations [8,9]. Within this framework, morphological assessment remains an essential approach for delineating taxonomic boundaries and for pinpointing populations that exhibit valuable attributes for both conservation initiatives and ornamental utilization [10]. Numerous investigations on diverse *Iris* taxa—such as *I. atropurpurea, I. germanica, I. histrio, I. hymenospatha, I. japonica, I. laevigata, I. maackii,* and *I. pseudacorus*—have affirmed that multivariate statistical techniques employing an extensive array of morphological parameters, especially those linked to floral traits like flower diameter, petal shape, and pigmentation, are effective in differentiating accessions and uncovering genetic structuring within wild populations [10–16].

Morphological characteristics, particularly floral attributes such as falls, standards, crests, and spathes, play a fundamental role in the taxonomic identification and breeding of *Iris* species [17–19]. In addition, the evaluation of quantitative traits including flower dimensions, floral shape parameters, and pigmentation features provides valuable information not only for phenotypic discrimination but also for understanding adaptive responses to varying environmental conditions [20]. In recent years, multivariate statistical techniques such as principal component analysis, cluster analysis, and regression-based approaches have become indispensable tools for exploring patterns of phenotypic variation and identifying relationships among morphological traits within natural plant populations [15].

Despite the ornamental and ecological importance of *I. persica*, detailed information regarding the extent of phenotypic variation within its natural populations remains limited. Therefore, the present study was undertaken to (i) assess the level of phenotypic diversity among wild *I. persica* accessions collected from different ecological regions of Markazi Province, Iran; (ii) determine the morphological traits that contribute most significantly to accession differentiation using multivariate analytical methods; and (iii) identify promising genotypes with potential value for ornamental breeding and germplasm conservation. The results provide new insights into the intraspecific diversity of *I. persica* and establish a valuable foundation for future taxonomic investigations, conservation planning, and breeding programs aimed at improving ornamental and adaptive characteristics.

## 2. Materials and methods

### 2.1. *Plant material*

**Fig 1:**
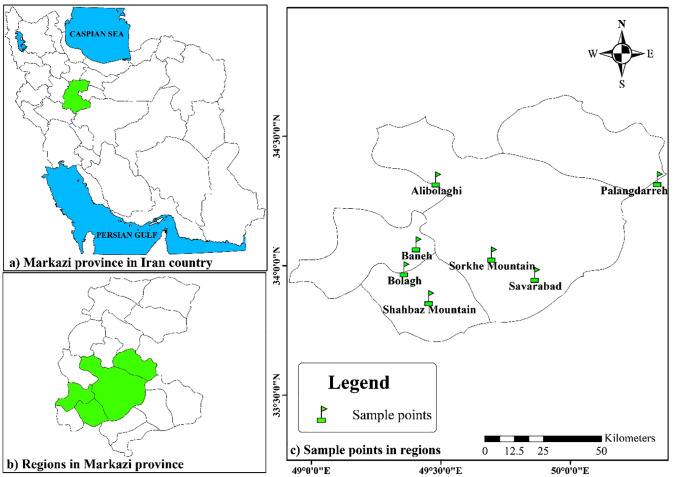
Geographic distribution of the 78 naturally occurring *Iris persica* accessions collected from seven ecogeographical regions of Markazi Province, Iran. The map was generated by the first author, Prof. Dr. Ali Khadivi, using ArcGIS software v10.1 (http://www.arcgis.com/home/item.html?id=30e5fe3149c34df1ba922e6f5bbf808f) and is original; no third-party copyrighted or proprietary material was used.

In May 2023, a comprehensive morphological characterization was performed on 78 naturally occurring accessions of *I. persica* collected from seven geographically distinct regions of Markazi Province, Iran. The sampled accessions were distributed as follows: Sorkhe (15 accessions), Shahbaz (15), Bolagh (10), Alibolaghi (10), Palangdarreh (10), Savarabad (10), and Baneh (8). The geographic distribution of the collection sites is presented in Fig 1. A location map was prepared using ArcGIS software version 10.1 [21]. The study region is characterized by warm and dry summers and semi-cold winters, frequently accompanied by snow cover.

To ensure representative sampling of the natural populations and to reduce the likelihood of collecting genetically identical individuals, sampled plants were selected at a minimum distance of 200 m from one another [22,23]. Each accession represented a single, independently growing individual, and one plant was evaluated per accession. Accordingly, each accession was treated as a single biological replicate, and all morphological measurements were recorded during the flowering stage. This sampling strategy was adopted to maximize the capture of phenotypic variation occurring within and among populations.

The taxonomic identification of the accessions was confirmed by Dr. Alireza Khaleghi. A representative voucher specimen (accession number IP-2314) has been deposited in the publicly accessible herbarium of the Faculty of Agriculture and Natural Resources, Arak University, Iran.

### Statement specifying permissions

For this study, we acquired permission to study *I. persica* issued by the Agricultural and Natural Resources Ministry of Iran. Inclusivity-in-global-research-questionnaire is provided in S1 File.

### Statement on experimental research and field studies on plants

The cultivated or wild-growing plants sampled comply with relevant institutional, national, and international guidelines and domestic legislation of Iran.

### 2.2. *Morphological evaluations*

A total of thirty-nine morphological traits pertaining to stem, leaf, and flower characteristics were evaluated. All morphological assessments were conducted at the time of flowering. Quantitative traits—including plant height, stem length, stem diameter, peduncle diameter, peduncle width, peduncle length, spathe width and length, flower diameter and length, fall width and length, crest width and length, standard width and length, anther length, and filament length—were precisely measured using a digital caliper.

The ratio of patch surface area to fall width was used to determine the proportional coverage of the signal patch on the fall. Similarly, the flower shape was calculated as the ratio of flower diameter to flower height, following the methodology of Sapir et al. [24]. Moreover, the flower surface area and patch surface area were calculated based on the specific equations proposed by Sapir et al. [24] (Eqs. 1 and 2).


$$\begin{array}{c} Flower\;surface\;=\;flower\;diameter\;\times \;flower\;height\;(in\;cm^{\text{2}}) \end{array}$$ (1)

$$\begin{array}{c} Patch\;surface\;=\;patch\;length\;\times \;width\;(in\;cm^{\text{2}}) \end{array}$$ (2)

Qualitative characteristics—including leaf shape, leaf color, flower scent, as well as the coloration of the fall, standard, and crest—were evaluated using standardized coding schemes, categorical scoring approaches, and structured rating scales to ensure consistency and reproducibility in the assessments. Morphological trait descriptors and scoring criteria were adopted from previously published studies on wild *Iris* germplasm characterization [22,23]. In addition, color evaluations were standardized using the Royal Horticultural Society (RHS) Colour Chart [RHS, 2015 25] under natural daylight conditions at midday, following the recommendations of McGuire [26], which emphasize the importance of consistent illumination, observer calibration, and the use of standardized color reference systems to minimize subjective bias and ensure objective and reproducible color determination across all accessions.

### 2.3. *Statistical analysis*

Descriptive statistics, including minimum, maximum, mean, standard deviation (SD), and coefficient of variation (CV), were calculated for all measured morphological traits using OriginPro^®^ 2025b software [27]. The relationships among the evaluated traits were further examined by calculating Pearson’s correlation coefficients (r) using the same statistical software. Principal component analysis was employed using the same software to identify the primary variables contributing to genotypic differentiation. To improve the interpretability of the principal components, Varimax rotation with Kaiser Normalization was applied, facilitating clearer interpretation of trait loadings on the principal components. For clustering purposes, hierarchical cluster analysis was performed using Ward’s linkage method and Euclidean distance, also implemented in OriginPro^®^ 2025b. Before cluster analysis, data preprocessing included min–max normalization (scaling data between 0 and 1) followed by Z-score standardization to ensure comparability across traits and to minimize potential biases due to differences in measurement scales [28]. A biplot based on the first two principal components (PC1 and PC2) was generated to visualize the spatial distribution of accessions and associated traits within a reduced-dimensional framework. Additionally, principal component (PC) scores were computed for each genotype, based on the principal component analysis model derived from *I. persica* accessions, to summarize multidimensional variation and to reveal patterns of structural differentiation among the accessions. Furthermore, to identify the traits exerting a significant effect on floral characteristics, which were considered as dependent variables, multiple linear regression analysis was performed. This analysis was conducted using the stepwise selection method in SPSS^®^ software (SPSS Inc., Chicago, IL, USA), following the analytical procedures described by Norusis [29], Efe et al. [30], and [31].

## 3. Results and discussion

### 3.1. *Descriptive statistics among accessions*

Descriptive data on the morphological traits of the examined *I. persica* accessions are presented in Table 1. Previous studies by Goodarzi et al. [32], Mostafa et al. [33], Elwakil et al. [34], and Khadivi et al. [35] reported that some variables exhibited no variation among accessions (CV = 0.00%) and were therefore excluded from further analyses. Similarly, in the present study, one variable, namely flower scent, showed no variation (CV = 0.00%) among the evaluated accessions and was consequently omitted from further analyses. Therefore, the remaining 38 morphological variables were retained for subsequent evaluations. The observed ranges and coefficients of variation indicated considerable phenotypic variability among the studied accessions. The traits showing the highest levels of phenotypic variation included standard color (CV = 68.12%), fall color (62.79%), crest color (61.61%), stigmatic lip color (60.84%), and leaf shape (60.66%). In contrast, the lowest coefficients of variation were recorded for fall length (11.55%), crest length (11.37%), stem diameter (11.03%), style arm length (10.77%), and patch length (10.29%). Out of the 38 assessed traits, 22 (representing 57.89%) exhibited coefficients of variation exceeding 20.00%, suggesting a high degree of genetic diversity among the accessions [36]. Traits with CV values above this threshold are considered highly polymorphic and are regarded as effective descriptors for differentiating between accessions, genotypes, or cultivars [37]. Furthermore, traits exhibiting broader quantitative ranges tend to correspond with higher CV values, making them particularly valuable for use in selection and breeding programs [38]. On the other hand, traits characterized by lower CV values reflect more uniform expression across accessions and indicate greater phenotypic stability, as also noted by Khadivi-Khub and Etemadi-Khah [37].

**Table 1:** Descriptive statistics and phenotypic variation of 38 morphological traits evaluated among 78 *Iris persica* accessions collected from natural populations.

Trait	Abbr	Unit	Min	Max	Mean	±SD	CV (%)
Plant length	V1	cm	6.03	12.59	8.18	1.30	15.87
Stem length	V2	cm	2.43	6.13	4.24	0.75	17.63
Stem diameter	V3	mm	2.20	3.44	2.83	0.31	11.03
Peduncle diameter	V4	mm	2.87	6.01	4.39	0.78	17.67
Leaf number	V5	Number	2	5	3.99	0.80	20.00
Bottommost leaf length	V6	mm	20.90	70.79	47.82	11.35	23.74
Bottommost leaf width	V7	mm	5.94	25.02	11.62	4.42	38.06
Leaf shape	V8	Code	1	5	1.97	1.20	60.66
Leaf color	V9	Code	1	5	4.26	1.29	30.38
Spathe length	V10	mm	25.86	64.56	39.17	7.96	20.33
Spathe width	V11	mm	6.20	25.66	15.54	4.19	26.97
Flower number	V12	Number	1	3	1.21	0.44	36.12
Flower diameter	V13	mm	34.52	80.56	53.53	8.86	16.56
Flower length	V14	mm	30.95	67.76	40.98	7.05	17.19
Flower diameter/length	V15	Ratio	0.86	1.65	1.32	0.18	13.90
Flower surface	V16	cm²	12.49	44.78	22.29	6.89	30.93
Fall length	V17	mm	35.39	57.70	45.21	5.22	11.55
Fall width	V18	mm	14.58	30.44	21.72	3.60	16.57
Fall color	V19	Code	1	8	3.44	2.16	62.79
Standard length	V20	mm	12.71	24.00	18.19	2.98	16.40
Standard width	V21	mm	2.67	8.38	5.75	1.29	22.50
Standard color	V22	Code	1	8	3.03	2.06	68.12
Crest length	V23	mm	30.54	53.95	41.41	4.71	11.37
Style arm length	V24	mm	22.60	37.05	28.75	3.10	10.77
Style crest length	V25	mm	7.65	18.01	12.66	2.59	20.48
Crest width	V26	mm	7.31	15.55	11.19	2.02	18.03
Crest color	V27	Code	1	7	3.05	1.88	61.61
Patch width	V28	mm	1.21	3.28	2.04	0.53	25.96
Patch length	V29	mm	29.83	47.87	37.12	3.82	10.29
Patch surface	V30	cm²	0.40	1.57	0.77	0.26	34.09
Patch surface/fall width	V31	Ratio	0.18	0.55	0.35	0.08	23.92
Patch color	V32	Code	1	7	4.82	1.17	24.27
Stigmatic lip length	V33	mm	1.64	6.87	2.70	0.94	34.75
Stigmatic lip color	V34	Code	1	9	4.15	2.53	60.84
Anther length	V35	mm	7.94	16.07	12.45	1.61	12.93
Anther color	V36	Code	1	3	1.82	0.99	54.40
Filament length	V37	mm	10.92	21.53	16.44	2.29	13.93
Filament color	V38	Code	1	5	3.21	1.00	31.09

*Abb*r Abbreviations, *Max* Maximum, *Min* Minimum, *± SD* Standard Deviation, *CV* Coefficient of Variation.

The quantitative range observed among the *I. persica* accessions demonstrated considerable variation in key morphological traits. Plant length ranged from 6.03 cm in the ‘Palangdarreh-6’ accession to 12.59 cm in ‘Sorkhe-6’. This range highlights substantial differences in vegetative growth habits, which may reflect genotypic variability or local environmental influences such as light availability or soil fertility. Stem length showed a minimum of 2.43 cm in ‘Palangdarreh-6’ and reached up to 6.13 cm in ‘Bolagh-9’. The variation in stem elongation may have implications for plant architecture and mechanical support, influencing overall plant vigor and adaptability. In terms of floral structures, spathe length varied between 25.86 mm in ‘Palangdarreh-6’ and 64.56 mm in ‘Shahbaz-11’. This wide span suggests potential differences in flower protection and development, possibly linked to microclimatic conditions or pollination strategies. Flower diameter exhibited a wide range, from 34.52 mm in ‘Palangdarreh-3’ to a maximum of 80.56 mm in ‘Sorkhe-12’. The significant variation in flower size is particularly relevant for ornamental value and may also impact pollinator attraction and reproductive efficiency. Similarly, flower length extended from 30.95 mm in ‘Baneh-2’ to 67.76 mm in ‘Sorkhe-11’. The elongated floral structures observed in certain accessions could indicate selection for visual traits or adaptation to specific pollination syndromes. Among the floral organs, fall length was lowest in ‘Palangdarreh-8’ (35.39 mm) and highest in ‘Shahbaz-11’ (57.70 mm), while fall width ranged from 14.58 mm in ‘Baneh-2’ to 30.44 mm in ‘Shahbaz-11’. These measurements show notable variation in petal morphology, which may be influenced by genetic factors regulating floral symmetry and attractiveness. The standard length varied between 12.71 mm in ‘Palangdarreh-8’ and 24.00 mm in ‘Bolagh-8’. This trait contributes to the overall flower profile and plays a role in the visual identity of the accession, which is significant for ornamental selection. Lastly, style crest length was recorded at 7.65 mm in ‘Palangdarreh-8’ and reached 18.01 mm in ‘Sorkhe-13’. Differences in this reproductive structure may influence pollination efficiency and reproductive isolation mechanisms. These findings collectively indicate a broad phenotypic spectrum, which may reflect underlying genotypic diversity and potential environmental interactions. The overall morphological variation provides a strong basis for identifying superior ornamental traits, understanding ecological adaptability, and informing conservation and breeding strategies in *I. persica*. The pattern of morphological variation observed in the present study aligns closely with findings reported in previous investigations on *Iris* taxa. Azimi et al. [39] emphasized substantial variability in plant architecture and floral morphology among Iranian *Iris* species, proposing these differences as valuable for both taxonomic delineation and the exploitation of native germplasm in breeding efforts. In a separate investigation, [40] employed multivariate analytical approaches to evaluate hybrids of *I. germanica*, successfully identifying floral traits with strong discriminatory power across genotypes, which they advocated as selection criteria in hybridization programs. Parallel observations were made by Asgari et al. [41], who documented pronounced morphological divergence among wild *Iris* species with ornamental potential, particularly highlighting floral dimensions as key determinants of aesthetic and horticultural value. Moreover, Ghorbani et al. [42] reported marked morphological differentiation within *I. pseudacorus* accessions, particularly in traits related to vegetative vigor and reproductive structures. These variations were interpreted as evidence of region-specific adaptation, with implications for both conservation biology and the development of locally adapted cultivars. Collectively, these studies underscore the relevance of morphological characterization in capturing the diversity and guiding the utilization of *Iris* germplasm for ornamental breeding and conservation strategies.

These findings collectively indicate a broad phenotypic spectrum among the *I. persica* accessions, reflecting not only underlying genotypic diversity but also potential environmental influences. The marked morphological and phenological variability observed across sites appears to represent adaptive responses to ecological heterogeneity within the species’ natural distribution range. In particular, variation in key floral traits—such as flower diameter, fall length, and spathe length—may be linked to differences in altitude, temperature regimes, and local pollinator assemblages, given that floral architecture often evolves under selective pressures imposed by microclimatic conditions and pollination ecology. Accessions from higher-altitude, cooler regions (e.g., Sorkhe and Shahbaz) exhibited larger floral structures and extended flowering periods, potentially enhancing pollinator attraction under shorter growing seasons, whereas those from lower-altitude, warmer and drier microhabitats tended to show more compact vegetative forms and smaller floral sizes, traits that may confer advantages for water-use efficiency and tolerance to heat or drought stress. These patterns underscore that the observed phenotypic differentiation in *I. persica* is shaped by both genetic and ecological factors, highlighting the adaptive significance of trait variability. Thus, integrating ecological context with phenotypic characterization provides deeper insight into population-level divergence and offers valuable guidance for habitat-specific conservation, germplasm management, and targeted ornamental breeding programs.

Based on the frequency distribution of qualitative morphological traits among the *I. persica* accessions presented in Table 2, a notable degree of phenotypic diversity was observed across several floral and vegetative characters. This variation reflects the species’ broad morphological plasticity and may hold significance for taxonomic differentiation, ecological adaptation, and ornamental breeding.

**Table 2:** Frequency distribution of qualitative vegetative and floral morphological traits among 78 naturally occurring *Iris persica* accessions.

Trait	Frequency (No. of accessions)		
	1	2	3
Leaf shape	Elongated (44)	–	Slightly Curved (30)
Leaf color	Green without white edge (7)	–	Dark green without white edge (15)
Fall color	White background + A very small number of purple spots (10)	White/Milky background + Pale purple veins (25)	White/Milky background + Purple veins (17)
Standard color	White (15)	White/Milky (30)	White + Pale purple veins (13)
Crest color	White/Milky background + Pale muddy green in middle (22)	White/Milky background + Muddy green in middle (16)	White/Milky background + Pale purple in middle (10)
Patch color	Yellow background + Pale purple spots (3)	–	Yellow background + Purple spots (8)
Stigmatic lip color	White/Milky (14)	–	White/Milky/Pale yellow (33)
Anther color	Pale yellow (46)	–	Yellow (32)
Filament color	White (6)	–	White/Milky (58)
Flower scent	Little (78)	–	–
**Trait**	**Frequency (No. of accessions)**		
	**4**	**5**	**6**
Leaf shape	–	Curved (4)	–
Leaf color	–	Dark green with white edge (56)	–
Fall color	Pale purple/Milky background + Pale purple veins (7)	Pale purple/Milky background + Purple veins (5)	Pale purple background + Pale purple veins (2)
Standard color	Milky (4)	Milky + Pale purple veins (4)	Milky + Purple veins (4)
Crest color	White/Milky background + Purple in middle (10)	Milky background + Pale purple in middle (10)	Milky/Purple background + Purple in middle (6)
Patch color	–	Yellow background + Dark purple spots (60)	–
Stigmatic lip color	–	Milky/Pale yellow (12)	–
Anther color	–	–	–
Filament color	–	Milky (14)	–
Flower scent	–	–	–
**Trait**	**Frequency (No. of accessions)**		
	**7**	**8**	**9**
Leaf shape	–	–	–
Leaf color	–	–	–
Fall color	Pale purple background + Purple veins (4)	Pale purple background + Dark purple veins (8)	–
Standard color	Milky/purple (2)	Pale purple + Purple veins (6)	–
Crest color	Pale purple background + Purple in middle (4)	–	–
Patch color	Yellow background + Black spots (7)	–	–
Stigmatic lip color	Milky/Yellow (10)	–	Pale yellow (9)
Anther color	–	–	–
Filament color	–	–	–
Flower scent	–	–	–

Among the vegetative traits, leaf shape was predominantly represented by the “Elongated” form (44 accessions), followed by “Slightly Curved” (30) and “Curved” (4), indicating that while linearity is typical for *I. persica*, moderate deviations in leaf curvature also exist. Leaf color showed significant variation, with the majority of accessions (56) exhibiting a “Dark green with white edge”, while fewer accessions displayed “Dark green without white edge” (15) or “Green without white edge” (7), suggesting that variegation is a frequent trait and may serve as a visually desirable feature in ornamental selection. Floral traits displayed pronounced variability. Fall color, a key ornamental attribute, spanned a wide range of patterns and pigmentation intensities. The most common categories were “White/Milky background + Pale purple veins” (25 accessions) and “White/Milky background + Purple veins” (17). In comparison, more intense pigmentation such as “Pale purple background + Dark purple veins” was observed in fewer cases (8 accessions), indicating a spectrum from subtle to vivid coloration. Similarly, standard color showed diverse expressions: “White/Milky” (30), “White” (15), and multicolored combinations such as “White + Pale purple veins” (13) and “Milky + Purple veins” (4), reflecting variation in both base tone and venation patterns. Crest color, another diagnostic floral trait, displayed notable heterogeneity. The most frequently recorded pattern was “White/Milky background + Pale muddy green in middle” (22 accessions), followed by various combinations involving muddy green or purple shades. The presence of accessions with “Milky/Purple background + Purple in middle” (6) and “Pale purple background + Purple in middle” (4) highlights the range of anthocyanin expression and its potential taxonomic relevance. Patch color was most commonly observed as “Yellow background + Dark purple spots” (60 accessions), making it one of the most uniform traits. However, a few accessions showed variation with “Yellow background + Purple spots” (8) or “Yellow background + Black spots” (7). Such contrasting pigmentation can enhance visual appeal and may be genetically stable markers for cultivar identification. For stigmatic lip color, most accessions exhibited light tones, such as “White/Milky/Pale yellow” (33), “Milky/Pale yellow” (12), and “Milky/Yellow” (10), with fewer accessions expressing pure “White/Milky” (14) or “Pale yellow” (9). This suggests relatively low diversity in this reproductive structure, possibly due to functional constraints in pollination biology. Anther color was fairly consistent across accessions, dominated by two color classes: “Pale yellow” (46) and “Yellow” (32). Similarly, filament color exhibited limited variability, with the majority showing “White/Milky” (58) and smaller groups exhibiting “Milky” (14) or “White” (6), indicating relative uniformity in androecial traits. Lastly, flower scent appeared in only one qualitative category (“Little”) across all 78 accessions, suggesting either very low olfactory variation within *I. persica* or a limitation in sensory perception or classification methodology. Although scent is often a significant trait in ornamental evaluation, its minimal variability here suggests it may play a limited ecological or aesthetic role in this species.

In summary, Table 2 illustrates that floral color traits—particularly fall, standard, crest, and patch coloration—represent the most polymorphic features among *I. persica* accessions. These traits are of particular value for ornamental breeding due to their aesthetic impact and ease of visual assessment. In contrast, reproductive and androecial traits such as stigmatic lip, anther, and filament color show greater stability, potentially reflecting selective pressures for reproductive consistency. The observed patterns of variation provide essential morphological markers for germplasm characterization, and when integrated with molecular or ecological data, can inform both conservation strategies and targeted breeding programs aimed at developing novel ornamental cultivars of *I. persica*. Furthermore, the diversity observed in floral pigmentation and vegetative traits may also reflect adaptations to distinct microhabitats, such as variation in altitude, soil type, light exposure, or pollinator assemblages across the natural distribution range of *I. persica*, highlighting the ecological significance of morphological differentiation within the species.

**Fig 2:**
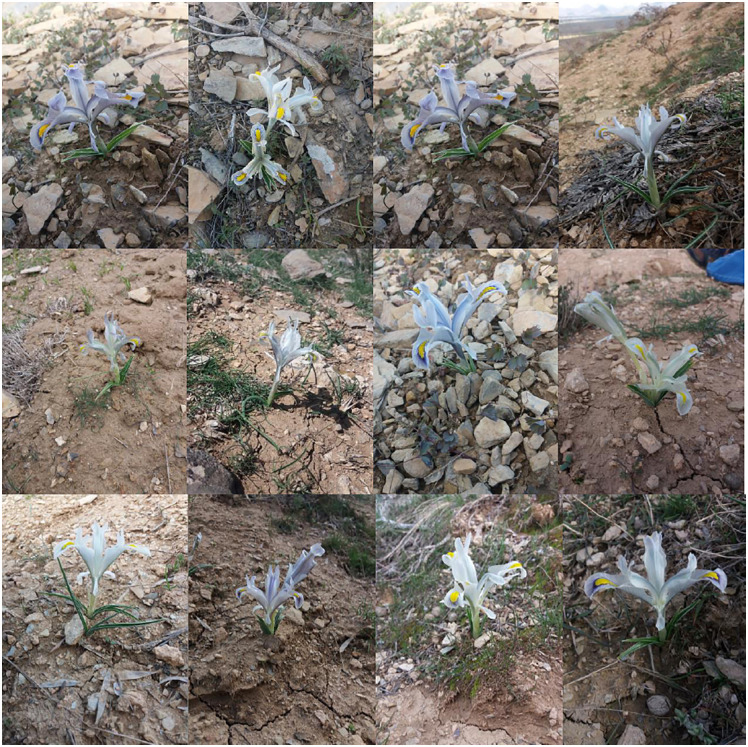
Variation in the bush of *Iris persica* accessions studied. These photographs were taken as part of the present study and are provided by the second author, Dr. Alireza Khaleghi, and are free from any third-party copyright restrictions.

**Fig 3:**
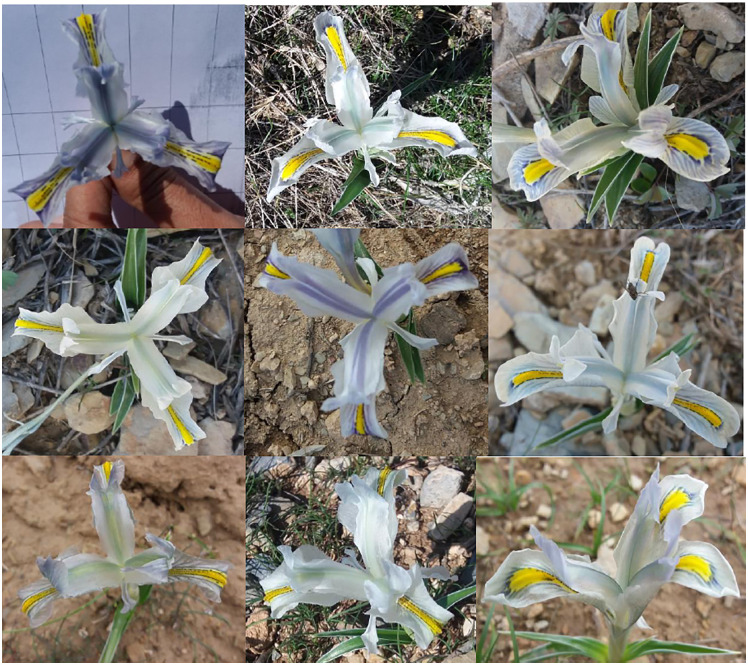
Variation in fall (sepal), standard (petal), and crest of *Iris persica* accessions studied. These photographs were taken as part of the present study and are provided by the second author, Dr. Alireza Khaleghi, and are free from any third-party copyright restrictions.

**Fig 4:**
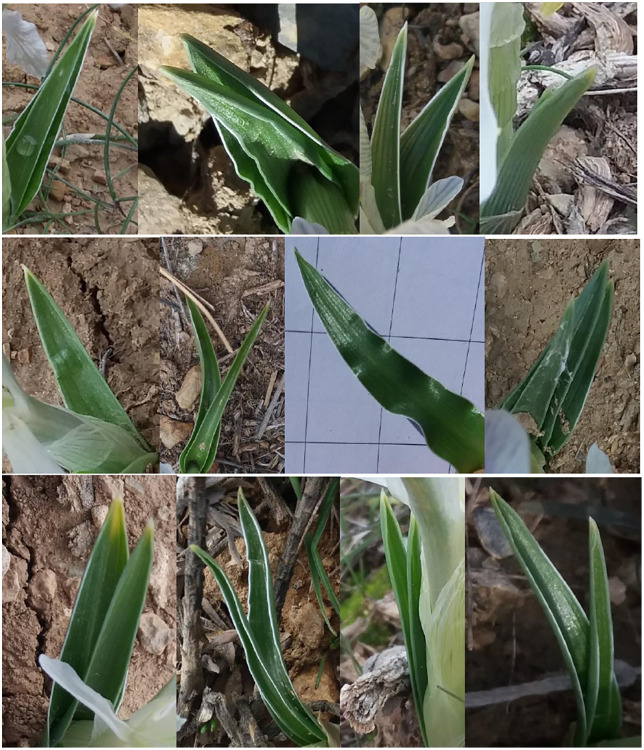
Variation in the leaves of *Iris persica* accessions studied. These photographs were taken as part of the present study and are provided by the second author, Dr. Alireza Khaleghi, and are free from any third-party copyright restrictions.

The morphological diversity observed among the examined *I. persica* accessions is illustrated in Fig 2, Fig 3, and Fig 4.

### 3.2. *Correlation matrix analysis (CMA)*

**Fig 5:**
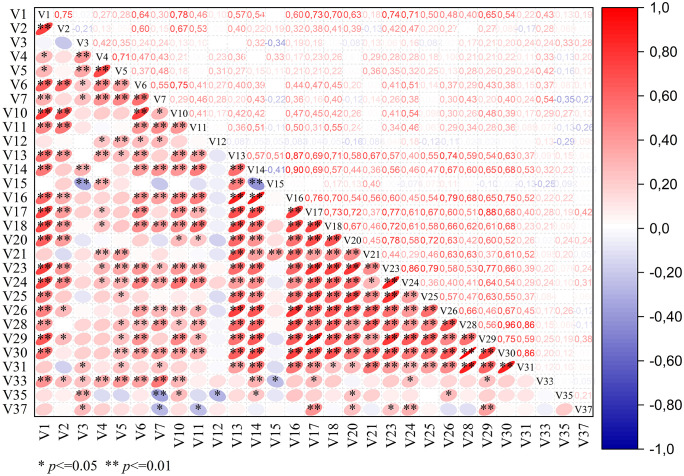
Pearson correlation matrix illustrating the relationships among quantitative morphological traits of *Iris persica* accessions. Positive and negative correlations are represented by color intensity and direction. For abbreviations, see Table 1.

As presented in Fig 5, the correlation matrix revealed several statistically significant associations among the quantitative morphological traits of *I. persica* accessions. A strong and highly significant positive correlation was detected between flower diameter and flower surface (*r* = 0.87, *p* < 0.01), indicating that larger floral diameters contribute substantially to overall floral area. This relationship underscores the integral role of radial expansion in determining floral display, which may influence pollinator attraction and visual prominence. Fall length was also significantly correlated with flower surface (*r* = 0.76, *p* < 0.01), standard length (*r* = 0.72, *p* < 0.01), and patch length (*r* = 0.88, *p* < 0.01). These correlations suggest a coordinated development of petal structures, where longer falls are often accompanied by proportionally longer standards and larger pigment patches, potentially enhancing floral symmetry and visual signaling. The particularly strong association between fall length and patch length may indicate genetic linkage or co-regulation of traits contributing to floral ornamentation. A significant and positive correlation was also found between flower diameter and flower length (*r* = 0.76, *p* < 0.01), supporting the idea that overall flower size scales consistently in both radial and longitudinal dimensions. This morphological consistency may reflect selective pressures favoring balanced floral architecture in natural populations or under cultivation. Additionally, spathe length exhibited a strong positive correlation with bottommost leaf length (*r* = 0.75, *p* < 0.01), suggesting a potential developmental coordination between vegetative and floral bract elongation. This association could be indicative of genotype-dependent growth patterns or resource allocation strategies. Moreover, spathe length showed moderate but significant correlations with fall width (*r* = 0.42, *p* < 0.01) and standard length (*r* = 0.26, *p* < 0.05), further supporting its integrative role within the floral architecture.

Collectively, these correlations demonstrate the presence of tightly interlinked morphological modules within *I. persica*, particularly among floral traits. Understanding such trait interdependencies is essential for selecting desirable combinations in ornamental breeding and for interpreting the evolutionary dynamics of flower morphology in the genus *Iris*.

Taken together, the pattern of correlations observed among both vegetative and reproductive traits highlights a degree of morphological integration that likely reflects shared genetic regulation and coordinated developmental pathways. These findings mirror the results of [40], who reported similar trait associations in *I. germanica* hybrids, and Asgari et al. [41], who emphasized the close interplay between floral dimensions and vegetative vigor in wild *Iris* species. The recurrence of such trait linkages across studies and taxa suggests that the integration of morphological features may be a conserved characteristic within the genus, contributing to both ecological adaptation and aesthetic value. As such, recognizing and quantifying these trait interactions not only provides a deeper understanding of phenotypic complexity in natural populations but also informs targeted breeding programs aiming to enhance ornamental performance under variable environmental conditions.

### 3.3. *Multiple regression analysis (MRA)*

To identify the most influential predictor variables affecting the dependent traits, a stepwise multiple linear regression analysis was conducted (Table 3). This method, implemented using the linear regression module of the statistical software, systematically added or removed variables based on a significance threshold of *p* ≤ 0.05. Through this iterative selection process, a refined and parsimonious model was generated, retaining only those explanatory variables that contributed significantly to the variation observed in the dependent trait, in agreement with the methodological framework outlined by Draper and Smith [43].

**Table 3:** Multiple regression analysis (MRA) identifying morphological traits significantly associated with flower-related characteristics in *Iris persica* accessions.

Dependent character	Independent character	*r*	*r^2^*	Adjusted *r^2^*	SEE	*F*-value	Sig.	*β*	*t*-value	*p*-value	Tolerance	VIF
Flower diameter	Flower surface	0.867ᵃ	0.75	0.749	4.44313	230.43	<0.001^a^	**0.81**	95.08	0.001*	0.212	4.711
	Flower diameter/length	0.993ᵇ	0.99	0.986	1.03943	2762	<0.001^b^	**0.41**	65.66	0.001*	0.397	2.519
	Peduncle diameter	0.996ᶜ	0.99	0.992	0.79855	3137	<0.001^c^	**0.13**	17.45	0.001*	0.266	3.766
	Standard length	0.997ᵈ	0.99	0.993	0.72485	2860	<0.001^d^	**0.07**	10.30	0.001*	0.307	3.254
	Patch surface	0.998ᵉ	1.00	0.996	0.56771	3740	<0.001^e^	**−0.04**	−5.80	0.001*	0.349	2.867
	Stem diameter	0.998ᶠ	1.00	0.996	0.56396	4737	<0.001^f^	−0.07	−9.70	0.001	0.34	2.945
	Bottommost leaf width	0.999ᵍ	1.00	0.997	0.50117	4803	<0.001^g^	−0.02	−2.64	0.01	0.188	5.325
	Stigmatic lip length	0.999ʰ	1.00	0.998	0.43586	5295	<0.001^h^	0.02	3.68	0.001	0.567	1.763
	Filament length	0.999ⁱ	1.00	0.998	0.4051	5256	<0.001^i^	0.03	5.23	0.001	0.523	1.913
	Bottommost leaf length	0.999ʲ	1.00	0.998	0.3894	4978	<0.001^j^	**−0.03**	−4.90	0.001*	0.399	2.507
	Anther length	0.999ᵏ	1.00	0.998	0.37881	4676	<0.001^k^	0.02	3.30	0.001	0.418	2.395
	Spathe width	0.999ˡ	1.00	0.998	0.36836	4451	<0.001^l^	**0.03**	4.58	0.001*	0.367	2.724
	Crest width	0.999ᵐ	1.00	0.998	0.35055	4469	<0.001^m^	**0.03**	3.09	0.001*	0.193	5.171
Fall length	Patch length	0.875ᵃ	0.77	0.763	2.54167	249.13	<0.001^a^	**0.36**	8.60	0.001*	0.209	4.774
	Flower diameter	0.912ᵇ	0.83	0.828	2.16757	186.02	<0.001^b^	**0.49**	9.66	0.001*	0.149	6.719
	Patch surface/fall width	0.926ᶜ	0.86	0.852	2.01243	148.21	<0.001^c^	**−0.66**	−11.66	0.001*	0.117	8.557
	Flower diameter/length	0.937ᵈ	0.88	0.872	1.86964	131.97	<0.001^d^	−0.36	−10.27	0.001	0.301	3.324
	Filament length	0.952ᵉ	0.91	0.901	1.64651	140.55	<0.001^e^	**0.26**	10.30	0.001*	0.608	1.644
	Patch surface	0.960ᶠ	0.92	0.915	1.51864	139.95	<0.001^f^	**0.80**	9.63	0.001*	0.054	18.439
	Stigmatic lip length	0.965ᵍ	0.93	0.924	1.43789	135.13	<0.001^g^	**0.24**	8.27	0.001*	0.46	2.174
	Bottommost leaf width	0.967ʰ	0.94	0.928	1.39993	125.34	<0.001^h^	−0.47	−10.62	0.001	0.196	5.091
	Anther length	0.969ⁱ	0.94	0.932	1.36314	118.04	<0.001^i^	−0.24	−9.01	0.001	0.518	1.932
	Crest width	0.973ʲ	0.95	0.939	1.28578	120.34	<0.001^j^	**0.10**	2.74	0.01*	0.27	3.7
	Bottommost leaf length	0.979ᵏ	0.96	0.952	1.13871	141.26	<0.001^k^	**0.18**	5.30	0.001*	0.331	3.018
	Plant length	0.979ˡ	0.96	0.953	1.1357	156.14	<0.001^l^	**−0.22**	−5.14	0.001*	0.213	4.688
Spathe length	Plant length	0.779ᵃ	0.61	0.602	5.02343	117.43	<0.001^a^	**0.94**	10.42	0.001*	0.261	3.832
	Style crest length	0.849ᵇ	0.72	0.713	4.2659	96.61	<0.001^b^	−0.17	−2.50	0.02	0.447	2.239
	Bottommost leaf length	0.885ᶜ	0.78	0.775	3.77912	89.26	<0.001^c^	**0.30**	4.76	0.001*	0.521	1.921
	Anther length	0.898ᵈ	0.81	0.796	3.5926	76.30	<0.001^d^	**0.20**	4.05	0.001*	0.882	1.134
	Stigmatic lip length	0.906ᵉ	0.82	0.809	3.48043	66.20	<0.001^e^	**−0.21**	−3.80	0.001*	0.666	1.501
	Standard length	0.922ᶠ	0.85	0.837	3.21267	66.99	<0.001^f^	−0.32	−3.67	0.001	0.282	3.543

Abbreviations: *r*, Pearson correlation coefficient; *r^2^*, coefficient of determination; Adjusted *r^2^*, adjusted coefficient of determination; SEE, standard error of estimate; *F*-value, F-statistic of the regression model; Sig., significance level of the regression model; *β*, standardized regression coefficient; *t*-value, Student’s t-statistic; *p*-value, probability value; VIF, variance inflation factor. Superscript letters (a–m) indicate successive steps of the stepwise multiple regression procedure and correspond to the models generated after the inclusion of each predictor variable.

*Multiple regression analysis correlations supported by the correlation matrix analysis.

Regarding flower diameter, the most influential predictor was flower surface (*β* = 0.81, *p* < 0.01), indicating that as the overall floral surface increases, so does the diameter of the flower. This is expected, as floral display size is inherently associated with surface expansion. Other positive contributors included the flower diameter-to-length ratio (*β* = 0.41), peduncle diameter (*β* = 0.13), and standard length (*β* = 0.07), all of which suggest that a robust floral architecture with wider and longer floral organs is associated with a larger flower diameter. Conversely, certain vegetative and reproductive traits showed negative relationships with flower diameter, such as patch surface (*β* = –0.04), stem diameter (*β* = –0.07), and bottommost leaf width (*β* = –0.02). This may reflect a trade-off in resource allocation between vegetative and reproductive growth, or developmental constraints that prevent simultaneous maximization of both vegetative bulk and floral size.

For fall length, patch surface emerged as the strongest positive predictor (*β* = 0.80), followed by flower diameter (*β* = 0.49) and filament length (*β* = 0.26), signifying a structural and functional integration among these traits. Interestingly, certain ratios and dimensions negatively influenced fall length, particularly the patch surface-to-fall width ratio (*β* = –0.66) and the flower diameter-to-length ratio (*β* = –0.36). These inverse associations may be interpreted as morphological compensation strategies, wherein an increase in one dimension leads to a proportional reduction in another to maintain structural balance. Bottommost leaf traits (width and length) also showed moderate effects (*β* = –0.47 and 0.18, respectively), suggesting that leaf size could indirectly impact floral elongation, possibly through developmental correlations or shared hormonal pathways.

Spathe length was almost exclusively driven by plant length (*β* = 0.94), demonstrating a strong morphological dependency between these two structural elements. Longer plants tend to develop longer spathes, likely as a supportive adaptation for floral display. Other notable predictors included bottommost leaf length (*β* = 0.30) and anther length (*β* = 0.20), whereas standard length (*β* = –0.32) and stigmatic lip length (*β* = –0.21) exerted significant negative influences. These contrasting effects again highlight the complex network of covariation and potential evolutionary trade-offs among floral organs in *I. persica*.

The observed associations underscore the intricate and multifaceted relationships among floral morphology, functional differentiation, and structural investment, indicating that both reproductive organ dimensions and morphological ratios jointly and, at times, antagonistically modulate spathe length. This interplay highlights the developmental coordination and potential evolutionary trade-offs inherent in the architectural design of *I. persica* flowers.

Collectively, the multivariate patterns revealed in this study point to a highly integrated morphological framework in *I. persica*, wherein floral traits exhibit strong interdependence with both generative and vegetative parameters. Such coordinated variation suggests that natural selection may act on specific trait constellations that collectively enhance reproductive performance, structural stability, and environmental adaptability. The detection of statistically robust and biologically meaningful predictors for primary floral traits provides a valuable foundation for future taxonomic refinement, ornamental cultivar development, and investigations into phenotypic plasticity within wild populations.

Among the diverse floral traits evaluated, flower diameter, fall length, and spathe length emerged as the most pivotal variables in determining overall floral architecture. Flower diameter represents a key aesthetic attribute, directly contributing to the ornamental appeal of the plant. Genotypes exhibiting larger floral diameters often demonstrate greater morphological variability and are thus particularly desirable in selection and breeding contexts. Furthermore, increased flower size is frequently associated with enhanced pollinator attraction, thereby promoting reproductive efficiency [18]. Fall length, corresponding to the length of the outer tepals, constitutes an important taxonomic character within the genus *Iris* and contributes significantly to the symmetry and visual balance of the flower. It may also function as a structural interface for pollinators during visitation, thereby affecting pollination dynamics [17]. Spathe length, which encapsulates and shields the floral bud before anthesis, is functionally relevant for protecting reproductive tissues and facilitating adaptation to varying environmental conditions. Its measurability at early developmental stages makes it a practical and informative trait for early selection decisions in breeding schemes [19].

### 3.4. *Principal component analysis (PCA)*

In line with Kaiser’s criterion, only those principal components (PCs) exhibiting eigenvalues greater than 1.0 were retained for further analysis, based on the rationale that components with eigenvalues below this threshold contribute less explanatory power than a single original variable and are therefore considered statistically negligible [44]. As a result, the first ten principal components—each surpassing the eigenvalue threshold of 1—were preserved, together accounting for 82.46% of the total observed variance. This outcome reflects a significant reduction in data dimensionality while effectively maintaining the majority of the original dataset’s informational content (Table 4).

**Table 4:** Eigenvalues, explained variance, and cumulative variance of principal components derived from morphological traits of *Iris persica* accessions.

Trait	Component									
	1	2	3	4	5	6	7	8	9	10
Plant length	0.64	0.17	0.05	**0.67ᵃ**	0.11	−0.01	0.09	−0.04	0.00	0.10
Stem length	0.33	0.14	−0.29	**0.79ᵃ**	−0.07	0.16	0.08	0.05	0.14	0.06
Stem diameter	−0.11	−0.03	0.27	0.03	0.58	−0.44	−0.39	−0.18	0.20	−0.12
Peduncle diameter	0.07	0.14	0.06	0.15	**0.84ᵃ**	0.26	0.11	−0.15	−0.03	−0.11
Leaf number	0.18	0.02	0.13	0.04	**0.86ᵃ**	0.07	0.19	0.03	0.01	0.12
Bottommost leaf length	0.13	0.22	0.13	**0.78ᵃ**	0.35	−0.03	0.07	−0.06	−0.01	0.00
Bottommost leaf width	−0.02	0.46	0.21	0.28	0.49	−0.28	0.31	0.34	0.00	−0.12
Leaf shape	−0.07	0.34	0.16	0.01	0.18	−0.36	0.01	0.51	−0.04	−0.42
Leaf color	−0.03	0.50	−0.21	0.08	−0.11	0.23	0.11	−0.38	0.26	−0.11
Spathe length	0.19	0.01	0.17	**0.88ᵃ**	0.10	−0.02	−0.04	−0.05	0.06	0.14
Spathe width	0.20	0.20	0.15	0.51	−0.10	−0.21	0.17	0.40	0.42	−0.26
Flower number	−0.17	0.04	0.08	0.16	0.26	−0.06	0.54	−0.21	0.08	0.22
Flower diameter	0.52	0.30	0.37	0.29	0.15	0.48	−0.21	0.14	0.13	−0.06
Flower length	0.48	0.35	0.45	0.26	0.04	−0.40	−0.26	0.23	0.10	0.08
Flower diameter/length	0.03	−0.02	−0.03	0.03	0.09	**0.95ᵃ**	0.04	−0.03	0.08	−0.14
Flower surface	0.52	0.38	0.49	0.32	0.08	0.02	−0.27	0.23	0.12	0.01
Fall length	**0.79ᵃ**	0.19	0.25	0.30	0.12	−0.08	−0.15	−0.19	0.00	−0.08
Fall width	**0.69ᵃ**	0.23	0.22	0.35	0.12	0.11	−0.03	0.23	0.04	−0.25
Fall color	0.22	**0.81ᵃ**	0.17	0.12	0.13	−0.02	0.03	0.17	0.04	0.07
Standard length	**0.85ᵃ**	0.08	0.13	0.12	−0.17	0.15	−0.13	−0.11	0.09	0.03
Standard width	0.38	0.14	0.55	−0.20	0.29	0.44	−0.23	0.11	0.11	0.10
Standard color	0.35	**0.78ᵃ**	0.20	−0.03	0.00	0.04	0.01	0.08	−0.08	0.06
Crest length	**0.86ᵃ**	0.17	0.19	0.19	0.15	−0.04	0.13	−0.13	0.17	0.05
Style arm length	0.58	0.16	0.18	0.40	0.18	−0.15	0.17	−0.24	0.33	0.00
Style crest length	**0.88ᵃ**	0.11	0.14	−0.12	0.06	0.11	0.02	0.06	−0.10	0.08
Crest width	0.52	0.36	0.43	0.19	0.00	0.19	−0.27	0.23	−0.02	0.03
Crest color	0.11	**0.79ᵃ**	0.12	0.05	0.13	−0.01	0.27	0.17	−0.02	0.11
Patch width	0.36	0.24	**0.83ᵃ**	0.09	0.10	0.04	0.06	0.16	−0.04	−0.05
Patch length	**0.71ᵃ**	0.04	0.41	0.27	0.12	−0.18	−0.03	−0.23	−0.01	−0.06
Patch surface	0.50	0.20	**0.79ᵃ**	0.18	0.11	−0.03	0.04	0.05	−0.04	−0.04
Patch surface/fall width	0.22	0.11	**0.89ᵃ**	−0.05	0.06	−0.10	0.05	−0.09	−0.07	0.11
Patch color	−0.11	0.07	0.09	−0.11	0.00	−0.10	−0.07	−0.01	**−0.88ᵃ**	−0.10
Stigmatic lip length	0.12	0.33	−0.12	0.27	0.50	−0.26	−0.04	0.08	−0.19	0.26
Stigmatic lip color	0.16	**0.71ᵃ**	0.17	0.25	0.08	−0.06	0.09	−0.05	−0.04	−0.13
Anther length	0.05	−0.16	0.17	0.24	−0.10	−0.02	**−0.78ᵃ**	−0.24	0.01	0.11
Anther color	−0.01	0.07	0.08	0.13	0.05	−0.14	0.09	0.06	0.07	**0.81ᵃ**
Filament length	0.24	−0.19	−0.02	0.06	0.16	−0.10	−0.07	**−0.78ᵃ**	−0.05	−0.11
Filament color	0.03	0.23	0.11	0.35	0.03	0.07	**0.69ᵃ**	−0.01	0.12	0.10
*Eigenvalue*	*12.65*	*3.83*	*3.31*	*2.65*	*2.35*	*1.64*	*1.47*	*1.29*	*1.10*	*1.04*
*Component degree of significance*	**	**	**	**	**	*	**	*	*	*
*Variance (%)*	*33.30*	*10.09*	*8.71*	*6.98*	*6.18*	*4.33*	*3.86*	*3.40*	*2.89*	*2.73*
*∑ variance (%)*	*33.30*	*43.39*	*52.09*	*59.07*	*65.25*	*69.58*	*73.43*	*76.84*	*79.72*	*82.46*

ᵃBold values represent variables with the highest absolute contribution (|loading|) to each principal component (PC) among significant eigenvalues (≥ 0.67). Level of significance for component loadings: *p < 0.05, **p < 0.01.

The first principal component (PC1), accounting for 33.30% of the total variation, was predominantly defined by floral structural attributes such as style crest length (0.88), crest length (0.86), standard length (0.85), fall length (0.79), patch length (0.71), and fall width (0.69). These high loading values suggest that PC1 reflects a comprehensive floral size and elongation axis, encompassing both reproductive and petaloid traits. The strong association of PC1 with traits related to floral architecture indicates that this component captures genotypic variability in overall flower morphology, particularly in dimensions affecting floral symmetry and visual prominence. The dominance of these traits in PC1 implies that selection pressures, whether natural or artificial, may primarily target this suite of characters in the context of pollinator attraction or ornamental breeding.

PC2, which explained 10.09% of the total variance, was mainly influenced by color traits, including fall color (0.81), crest color (0.79), standard color (0.78), and stigmatic lip color (0.71). This component represents a distinct axis of chromatic differentiation, reflecting variation in floral pigmentation. The concentration of color-related loadings in PC2 indicates that floral coloration may be governed by independent genetic or environmental factors compared to structural traits. These findings underscore the importance of floral color as a separate but complementary dimension of phenotypic diversity, potentially playing a central role in pollinator specificity, ecological adaptation, and taxonomic delimitation.

The third principal component (PC3), contributing 8.71% to the total variation, was characterized by traits associated with the patch region, specifically patch surface/fall width ratio (0.89), patch width (0.83), and patch surface (0.79). This suggests that PC3 captures localized variation in the ornamental and functional aspects of the patch area, which may be critical for species recognition or pollination efficiency. The high contribution of patch morphology to this component implies a finer-scale structural diversification within *I. persica* populations, possibly reflecting ecological microadaptation or intra-populational polymorphism.

Taken together, the first three principal components accounted for 52.09% of the total morphological variation, indicating that a substantial proportion of trait diversity in *I. persica* can be explained by a limited number of trait combinations.

Analogous findings have been consistently documented in prior multivariate analyses of *Iris* species, further substantiating the robustness and relevance of the present study. For example, comprehensive morphometric evaluations conducted by Sapir et al. [24] in Israel, Azimi et al. [39] and Ghorbani et al. [42] in Iran, Bo et al. [45] in China, and Boltenkov et al. [46] in Russia reported that the first three principal components cumulatively accounted for over 40% of the total morphological variance. The convergence of these results across diverse ecological regions and taxonomic contexts not only lends empirical support to the patterns identified herein but also highlights the pervasive and taxonomically informative nature of principal component structures in *Iris*. This alignment reinforces the broader applicability of PCA as a reliable tool for elucidating complex trait interactions and phenotypic structuring within the genus.

**Fig 6:**
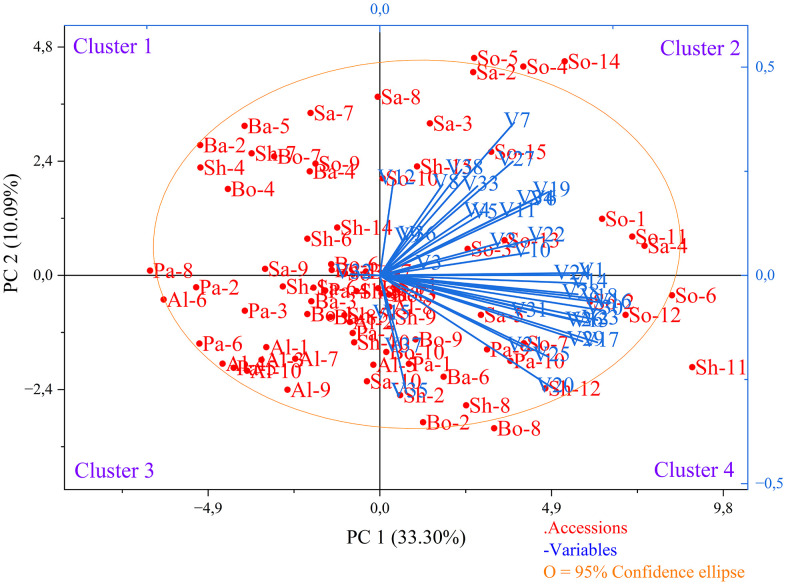
Principal component analysis (PCA) biplot showing the distribution of *Iris persica* accessions based on morphological traits. To improve readability, accession names were abbreviated as follows: So = Sorkhe, Sh = Shahbaz, Bo = Bolagh, Al = Alibolaghi, Pa = Palangdarreh, Sa = Savarabad, and Ba = Baneh. The accompanying numbers correspond to the original genotype codes assigned to each accession. For variable abbreviations, see Table 1.

The principal component biplot (PC1 vs. PC2), which encapsulates the multivariate morphological relationships among *I. persica* accessions, revealed that the majority of accessions were concentrated within the 95% confidence ellipse. This distribution pattern suggests a high degree of overall morphological similarity among most individuals analyzed. Nonetheless, several accessions—namely ‘Alibolaghi-6’, ‘Bolagh-8’, ‘Palangdarreh-8’, ‘Sorkhe-4’, ‘Sorkhe-5’, ‘Sorkhe-6’, ‘Sorkhe-11’, and ‘Sorkhe-14’—were positioned outside this confidence boundary, indicating that they deviate significantly from the central morphological cluster (Fig 6). The separation of these outlier accessions along the principal axes is likely driven by pronounced differences in one or more key morphological traits—such as floral dimensions, leaf configuration, or overall plant height—which exhibited strong loading values on PC1 and PC2. Their distinct placement in the biplot may reflect underlying genetic divergence or localized adaptation to unique microhabitat conditions within the species’ natural distribution [47,48]. The detection of these morphologically divergent genotypes holds particular significance for breeding programs, conservation efforts, and taxonomic classification. Their unique phenotypic profiles may encompass traits of ecological relevance or ornamental potential, thereby offering valuable resources for both applied and fundamental botanical research.

The principal component (PC) scores for *I. persica* accessions exhibited a wide range, extending from –6.39 for ‘Savarabad-1’ to 20.11 for ‘Alibolaghi-6’, thereby reflecting substantial multivariate morphological diversity among the evaluated accessions (Table 5). The accessions with the highest PC values included ‘Alibolaghi-6’ (20.11), ‘Sorkhe-4’ (19.26), ‘Sorkhe-14’ (17.88), ‘Shahbaz-11’ (17.73), ‘Sorkhe-11’ (17.68), ‘Sorkhe-12’ (16.62), ‘Sorkhe-6’ (16.43), ‘Palangdarreh-8’ (16.15), ‘Sorkhe-5’ (13.37), and ‘Bolagh-8’ (8.12). Notably, a subset of these accessions—namely ‘Alibolaghi-6’, ‘Bolagh-8’, ‘Palangdarreh-8’, ‘Sorkhe-4’, ‘Sorkhe-5’, ‘Sorkhe-6’, ‘Sorkhe-11’, and ‘Sorkhe-14’—fell outside the 95% confidence ellipse in the PCA biplot, indicating marked deviations in morphological traits relative to the main cluster.

**Table 5:** Principal component scores of 78 *Iris persica* accessions based on multivariate analysis of morphological traits.

Accession	PC1	PC2	PC3	PC4	PC5	PC6	PC7	PC8	Composite score
‘Sorkhe-1’	0.50	−0.14	0.65	1.52	−0.23	−0.23	1.58	0.77	4.41
‘Sorkhe-2’	−0.47	0.54	−0.46	−0.47	0.24	−1.91	−1.73	−0.56	−4.81
‘Sorkhe-3’	−1.01	0.31	−0.91	−1.41	1.47	−0.23	0.07	−1.43	−3.14
‘Sorkhe-4’	1.59	2.72	1.75	3.26	1.58	1.38	2.38	4.61	19.26
‘Sorkhe-5’	1.67	1.82	3.63	1.06	2.60	0.14	0.25	2.20	13.37
‘Sorkhe-6’	3.44	1.68	1.89	1.80	0.83	2.16	1.56	3.08	16.43
‘Sorkhe-7’	0.34	−1.76	0.32	−0.39	−0.68	0.61	1.03	0.93	0.42
‘Sorkhe-8’	−0.84	−0.31	0.33	0.98	−0.48	−0.19	−1.11	−1.20	−2.81
‘Sorkhe-9’	0.81	1.36	−0.07	1.00	0.36	−0.65	0.36	1.54	4.72
‘Sorkhe-10’	−0.04	1.57	−2.62	0.82	0.09	−0.30	0.09	−1.99	−2.38
‘Sorkhe-11’	2.39	2.54	3.32	1.66	0.90	1.26	3.16	2.46	17.68
‘Sorkhe-12’	2.34	2.56	2.47	3.42	0.82	2.17	1.38	1.48	16.62
‘Sorkhe-13’	0.30	0.26	0.01	−0.24	−1.42	−0.42	−0.34	−0.80	−2.65
‘Sorkhe-14’	1.56	2.53	3.51	3.17	2.51	2.32	−0.32	2.60	17.88
‘Sorkhe-15’	0.06	2.46	−0.19	0.30	−0.04	−1.17	1.14	0.75	3.32
‘Shahbaz-1’	0.79	−0.91	1.40	−1.40	0.59	2.19	−0.99	−0.57	1.10
‘Shahbaz-2’	0.10	−0.50	−1.55	0.07	−1.06	0.47	−0.92	1.55	−1.84
‘Shahbaz-3’	−0.78	−0.32	0.81	−1.23	0.23	1.31	−1.61	0.19	−1.41
‘Shahbaz-4’	0.26	0.78	−1.24	−1.32	0.52	0.30	0.25	0.35	−0.10
‘Shahbaz-5’	−0.68	0.23	0.29	−0.71	1.87	0.47	−1.19	0.66	0.94
‘Shahbaz-6’	−0.98	0.79	1.16	−0.82	0.96	0.41	0.82	1.90	4.25
‘Shahbaz-7’	−0.25	−0.75	−0.89	−0.82	−0.08	0.34	0.28	0.83	−1.34
‘Shahbaz-8’	0.01	1.45	−0.27	2.72	0.63	−0.86	−1.07	0.48	3.10
‘Shahbaz-9’	−0.22	0.71	0.47	−0.07	−0.85	−1.52	−0.45	0.86	−1.06
‘Shahbaz-10’	0.21	−1.25	0.17	0.39	−0.88	0.15	0.06	−1.14	−2.29
‘Shahbaz-11’	2.17	3.41	2.89	2.84	0.18	1.27	2.86	2.11	17.73
‘Shahbaz-12’	0.90	0.31	0.81	0.63	−0.83	−0.56	0.75	0.61	2.62
‘Shahbaz-13’	−0.77	−0.24	−0.49	0.08	2.32	−1.87	0.69	−1.61	−1.89
‘Shahbaz-14’	−0.47	1.09	0.06	−1.08	−0.72	0.68	−0.73	0.22	−0.95
‘Shahbaz-15’	0.05	−0.65	2.14	0.63	−2.03	0.19	−0.66	0.85	0.52
‘Bolagh-1’	−0.79	−0.12	0.51	0.87	−1.20	−0.34	−0.48	−0.65	−2.20
‘Bolagh-2’	1.77	0.41	−1.26	0.92	2.12	1.03	−1.52	−0.48	2.98
‘Bolagh-3’	1.27	−0.71	0.44	0.78	−0.93	−0.06	−3.24	−1.02	−3.47
‘Bolagh-4’	−0.25	−1.25	1.63	−1.43	−0.44	0.13	1.44	−1.44	−1.60
‘Bolagh-5’	1.16	0.01	−0.98	0.46	0.20	−0.60	0.07	−0.39	−0.06
‘Bolagh-6’	0.11	0.66	1.59	−1.24	2.13	−1.95	−0.15	0.59	1.74
‘Bolagh-7’	0.28	−0.62	−0.21	−0.49	−0.59	0.85	0.36	−0.69	−1.12
‘Bolagh-8’	0.52	3.85	0.57	1.14	0.95	0.65	−0.32	0.76	8.12
‘Bolagh-9’	−0.02	0.12	1.28	−0.59	0.55	−0.20	−0.22	1.10	2.01
‘Bolagh-10’	0.83	0.81	1.31	0.02	0.68	−0.31	0.32	−0.13	3.53
‘Alibolaghi-1’	0.10	0.60	−0.82	2.09	−1.01	−1.21	1.16	0.79	1.70
‘Alibolaghi-2’	0.62	0.63	−0.01	−0.90	0.08	−0.68	0.98	−0.15	0.57
‘Alibolaghi-3’	−0.83	−0.32	0.41	−0.56	−0.82	0.24	0.25	−0.51	−2.14
‘Alibolaghi-4’	−0.47	0.23	−1.45	−1.41	−0.72	−0.21	0.31	1.48	−2.24
‘Alibolaghi-5’	0.86	−0.16	−0.02	−1.00	−0.02	−0.29	0.32	−0.83	−1.14
‘Alibolaghi-6’	3.18	3.71	2.18	2.56	2.79	1.94	1.96	1.79	20.11
‘Alibolaghi-7’	0.10	−0.77	0.03	0.50	1.45	0.96	2.15	−0.77	3.64
‘Alibolaghi-8’	0.87	0.18	2.19	−0.81	−0.84	−0.60	−2.12	−0.53	−1.65
‘Alibolaghi-9’	−0.76	0.15	0.34	1.88	0.95	−0.58	−0.90	0.49	1.58
‘Alibolaghi-10’	−1.32	1.83	1.18	−0.47	−1.71	1.35	−0.12	1.24	1.99
‘Palangdarreh-1’	−1.59	−0.60	0.01	0.05	−0.45	0.62	−1.07	−0.14	−3.18
‘Palangdarreh-2’	0.12	0.51	0.71	−1.13	−1.53	1.28	0.33	−0.75	−0.45
‘Palangdarreh-3’	1.55	0.12	1.18	0.07	2.06	1.76	−0.25	0.97	7.45
‘Palangdarreh-4’	0.65	1.37	−0.97	0.69	1.06	−1.76	−1.18	−2.04	−2.19
‘Palangdarreh-5’	−0.27	0.72	1.50	0.07	1.63	−1.38	−1.70	−0.06	0.52
‘Palangdarreh-6’	0.38	−0.03	−2.07	−0.09	−1.30	0.67	0.37	−0.94	−3.01
‘Palangdarreh-7’	−0.51	−1.06	−0.06	0.96	−0.99	0.50	−0.53	−0.79	−2.49
‘Palangdarreh-8’	2.69	1.88	1.51	0.71	4.43	2.15	0.84	1.95	16.15
‘Palangdarreh-9’	−0.11	−0.22	0.61	0.76	−0.53	−0.58	−0.28	−2.30	−2.65
‘Palangdarreh-10’	−1.52	1.37	1.65	−0.25	0.58	0.31	3.08	1.12	6.34
‘Savarabad-1’	−0.13	−0.96	−1.61	0.20	−0.76	−1.42	−0.65	−1.08	−6.39
‘Savarabad-2’	1.69	0.88	−0.01	1.48	0.08	−0.86	1.52	0.54	5.32
‘Savarabad-3’	−1.04	−0.19	−0.88	−1.38	0.93	1.91	−1.40	0.56	−1.49
‘Savarabad-4’	−0.65	−0.49	−0.59	−0.86	0.05	−0.83	0.27	−0.05	−3.16
‘Savarabad-5’	−0.24	−0.91	−0.58	0.76	0.50	−0.98	0.10	0.75	−0.60
‘Savarabad-6’	−1.67	0.54	−0.66	0.57	−0.76	−1.81	−1.63	0.05	−5.37
‘Savarabad-7’	0.26	−0.90	0.64	−1.66	−0.07	−1.21	−0.65	0.05	−3.55
‘Savarabad-8’	−0.86	−0.39	1.01	−0.58	0.84	−1.13	0.53	1.44	0.86
‘Savarabad-9’	−2.47	−0.80	0.58	−0.20	0.37	−0.60	0.09	−0.16	−3.20
‘Savarabad-10’	1.17	0.25	0.34	−0.41	−0.49	−0.43	0.39	−0.42	0.40
‘Baneh-1’	0.29	2.08	0.87	−0.33	1.20	−0.41	−2.04	−1.01	0.66
‘Baneh-2’	−1.87	−0.35	0.02	1.68	0.33	−0.22	0.83	−2.21	−1.80
‘Baneh-3’	0.24	0.77	−1.48	1.14	0.34	−0.42	0.63	2.27	3.50
‘Baneh-4’	0.18	0.25	−0.46	−0.85	0.83	−0.86	0.07	−0.48	−1.31
‘Baneh-5’	0.48	0.33	1.04	−0.51	−0.27	−0.98	−0.44	0.38	0.03
‘Baneh-6’	0.76	−0.92	0.87	1.36	0.41	1.88	−0.77	−1.25	2.33
‘Baneh-7’	−1.78	1.50	0.65	−0.06	0.28	−1.13	2.45	0.13	2.05
‘Baneh-8’	0.11	0.73	0.48	0.22	−0.79	0.47	1.88	1.35	4.45

In addition to their distinct PC positions, certain accessions displayed unique trait values: for instance, ‘Sorkhe-12’ had the largest flower diameter at 80.56 mm, while ‘Shahbaz-11’ stood out with the longest fall length (57.70 mm) and the greatest spathe length (64.56 mm). These outcomes derived from the PC score analysis are in agreement with patterns observed in the descriptive statistics and PCA visualization, collectively affirming the pronounced morphological heterogeneity present among the accessions. In contrast to earlier research, which lacked explicit evaluation of PC scores, the present study’s incorporation of this analytical dimension has enhanced the depth and reliability of morphological differentiation among accessions.

### 3.5. *Hierarchical cluster analysis (HCA)*

**Fig 7:**
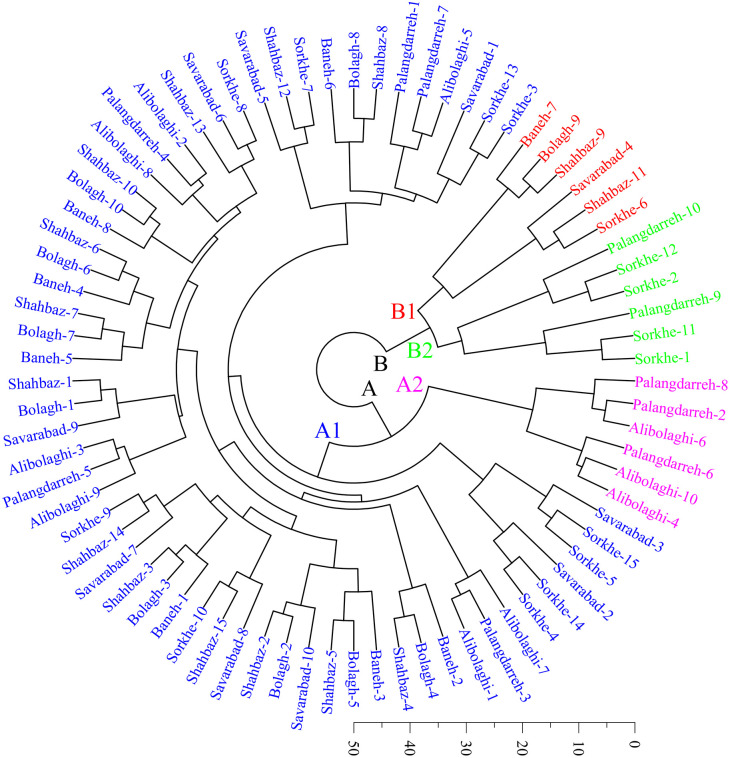
Hierarchical cluster dendrogram showing phenotypic relationships and grouping patterns among 78 *Iris persica* accessions based on morphological traits.

Fig 7 presents the dendrogram obtained via hierarchical cluster analysis (HCA), constructed using quantitative morphological traits of *I. persica* accessions. The complete step-by-step clustering information, based on Euclidean distance metrics, is provided in S1 Table. Collectively, these results offer a comprehensive overview of the morphological diversity and potential underlying genetic structuring among the 78 wild accessions sampled from the Markazi region. The dendrogram reveals a clear hierarchical grouping pattern, where accessions are progressively clustered into broader assemblages as linkage distance increases. This structure captures varying levels of phenotypic differentiation, with accessions clustered at shorter distances reflecting greater morphological similarity.

For instance, in the earliest stages of clustering, accessions ‘Shahbaz-3’ and ‘Bolagh-3’ were joined at a minimal Euclidean distance of 4.29, signifying a high degree of morphological congruence. As clustering advanced, the linkage distances progressively widened, culminating in a maximum fusion distance of 43.36 at the final stage, where ‘Sorkhe-1’ and ‘Sorkhe-3’—the most morphologically divergent accessions—were finally grouped. The wide range of linkage distances observed (4.29–43.36) underscores the considerable morphological variability present within the studied population.

Noteworthy is the pronounced increase in linkage distance during the terminal clustering stages (e.g., stages 76 and 77), suggesting the amalgamation of well-separated clusters. Such patterns may reflect the existence of distinct morphotypes or genetically structured subpopulations. Accessions that were merged in these later stages, such as ‘Sorkhe-3’ and ‘Sorkhe-4’, exhibit remarkable morphological distinctiveness and are likely candidates for further ecological, physiological, or molecular characterization. The final clustering of ‘Sorkhe-1’ and ‘Sorkhe-3’ at the highest distance provides additional support for their phenotypic uniqueness within the dataset.

In summary, the hierarchical clustering results highlight extensive intraspecific morphological divergence among *I. persica* accessions, which appear to be organized into both morphologically cohesive groups and highly differentiated individuals. This observed phenotypic structure indicates a broad spectrum of genetic diversity within the Markazi population, potentially shaped by environmental heterogeneity, spatial isolation, and localized selection pressures. These findings are of particular relevance for taxonomic classification, ecological assessment, and the formulation of targeted conservation and breeding strategies, especially for preserving genetically and morphologically distinct genotypes.

Cluster-based analytical approaches have been extensively utilized in prior research to investigate morphological variation and genetic structuring within *Iris* species. For instance, Azimi et al. [39] implemented HCA to delineate distinct phenotypic groups among Iranian *Iris* taxa, reflecting potential underlying genetic divergence. In a later investigation, [40] employed a combination of multivariate statistical techniques, including HCA, to categorize hybrids of *I. germanica*, thereby enhancing the selection process in breeding programs. Likewise, Asgari et al. [41] applied HCA to assess morphological diversity across wild *Iris* species with ornamental potential, underscoring the utility of this method in identifying phenotypically superior genotypes. In a related study, Ghorbani et al. [42] used clustering techniques to evaluate morphological differentiation among *I. pseudacorus* accessions, successfully identifying distinct ecotypes with possible relevance for conservation efforts.

## 4. Conclusions

This study provided a comprehensive morphological assessment of 78 wild *I. persica* accessions, revealing substantial phenotypic diversity across floral and vegetative traits. The observed variability, especially in floral dimensions (e.g., flower diameter, fall length, and spathe length) and color traits (fall, standard, crest, patch), underscores the species’ ornamental potential and ecological adaptability. PCA highlighted the significance of floral architecture and pigmentation as the primary axes of variation, while multiple regression analysis identified key predictors influencing floral trait development.

The identification of morphologically superior accessions—‘Alibolaghi-6’, ‘Sorkhe-4’, ‘Sorkhe-14’, ‘Shahbaz-11’, ‘Sorkhe-11’, ‘Sorkhe-12’, ‘Sorkhe-6’, ‘Palangdarreh-8’, ‘Sorkhe-5’, and ‘Bolagh-8’—presents valuable opportunities for the development of novel ornamental cultivars. Furthermore, the tight correlations observed among floral traits point to coordinated developmental pathways, suggesting that selection for one aesthetic feature may inadvertently enhance others. The presence of outlier genotypes outside the morphological core cluster suggests possible genetic divergence or localized adaptation, warranting further investigation through molecular and ecological studies.

Overall, the findings emphasize the importance of preserving wild *I. persica* germplasm and integrating its considerable morphological diversity into future breeding, selection, and conservation strategies. Although multi-season replication would undoubtedly strengthen inference regarding year effects and potential genotype × environment (G × E) interactions, the present single-season cross-sectional design provides a valid and scientifically robust basis for detecting among-accession phenotypic heterogeneity under uniform field conditions. To further enhance the reliability and broader applicability of these findings, future research should incorporate multi-year evaluations, complemented by molecular marker-based analyses and environmental covariates, to more comprehensively elucidate the genetic architecture trait variability and to support long-term conservation and horticultural utilization of this taxonomically and economically valuable species.

## Supporting information

S1 File Inclusivity-in-global-research-questionnaire. (DOCX)

S1 Table Cluster formation stages of *I. persica* accessions. (DOCX)
