# Supplementary material for: Phenotypic variations of naturally grown Iris persica L. accessions as revealed using multivariate analytical methods
Source: PLoS One. 2026 Jul 27;21(7):e0354156. doi: 10.1371/journal.pone.0354156 (PMC13405088; doi:10.1371/journal.pone.0354156)
Supplement: S1 Table — (DOCX) [file pone.0354156.s002.docx]

**S1 Table.** Cluster formation stages of *I. persica* accessions.

| Accession | Stage | Distance | Cluster1 | Cluster2 | Number of Clusters | New Cluster | Next Stage |
| --- | --- | --- | --- | --- | --- | --- | --- |
| ‘Sorkhe-1’ | 1 | 4.29 | 18 | 33 | 77 | 18 | 26 |
| ‘Sorkhe-2’ | 2 | 4.58 | 25 | 40 | 76 | 25 | 28 |
| ‘Sorkhe-3’ | 3 | 4.62 | 46 | 52 | 75 | 46 | 32 |
| ‘Sorkhe-4’ | 4 | 4.65 | 22 | 37 | 74 | 22 | 41 |
| ‘Sorkhe-5’ | 5 | 4.66 | 24 | 39 | 73 | 24 | 34 |
| ‘Sorkhe-6’ | 6 | 4.72 | 17 | 32 | 72 | 17 | 31 |
| ‘Sorkhe-7’ | 7 | 4.81 | 16 | 31 | 71 | 16 | 33 |
| ‘Sorkhe-8’ | 8 | 5.11 | 19 | 34 | 70 | 19 | 46 |
| ‘Sorkhe-9’ | 9 | 5.22 | 21 | 36 | 69 | 21 | 38 |
| ‘Sorkhe-10’ | 10 | 5.26 | 23 | 38 | 68 | 23 | 45 |
| ‘Sorkhe-11’ | 11 | 5.29 | 20 | 35 | 67 | 20 | 48 |
| ‘Sorkhe-12’ | 12 | 5.39 | 43 | 55 | 66 | 43 | 30 |
| ‘Sorkhe-13’ | 13 | 5.44 | 10 | 30 | 65 | 10 | 39 |
| ‘Sorkhe-14’ | 14 | 5.52 | 3 | 13 | 64 | 3 | 50 |
| ‘Sorkhe-15’ | 15 | 5.54 | 4 | 14 | 63 | 4 | 49 |
| ‘Shahbaz-1’ | 16 | 5.65 | 41 | 53 | 62 | 41 | 36 |
| ‘Shahbaz-2’ | 17 | 5.66 | 8 | 66 | 61 | 8 | 40 |
| ‘Shahbaz-3’ | 18 | 5.70 | 1 | 11 | 60 | 1 | 57 |
| ‘Shahbaz-4’ | 19 | 5.70 | 9 | 29 | 59 | 9 | 37 |
| ‘Shahbaz-5’ | 20 | 5.81 | 44 | 50 | 58 | 44 | 29 |
| ‘Shahbaz-6’ | 21 | 5.90 | 7 | 27 | 57 | 7 | 42 |
| ‘Shahbaz-7’ | 22 | 5.95 | 5 | 15 | 56 | 5 | 43 |
| ‘Shahbaz-8’ | 23 | 5.99 | 2 | 12 | 55 | 2 | 52 |
| ‘Shahbaz-9’ | 24 | 6.20 | 45 | 57 | 54 | 45 | 44 |
| ‘Shahbaz-10’ | 25 | 6.27 | 42 | 54 | 53 | 42 | 35 |
| ‘Shahbaz-11’ | 26 | 6.72 | 18 | 71 | 52 | 18 | 55 |
| ‘Shahbaz-12’ | 27 | 6.78 | 6 | 26 | 51 | 6 | 47 |
| ‘Shahbaz-13’ | 28 | 6.83 | 25 | 78 | 50 | 25 | 56 |
| ‘Shahbaz-14’ | 29 | 6.89 | 44 | 56 | 49 | 44 | 61 |
| ‘Shahbaz-15’ | 30 | 6.96 | 43 | 49 | 48 | 43 | 62 |
| ‘Bolagh-1’ | 31 | 6.99 | 17 | 70 | 47 | 17 | 54 |
| ‘Bolagh-2’ | 32 | 7.03 | 46 | 58 | 46 | 46 | 61 |
| ‘Bolagh-3’ | 33 | 7.45 | 16 | 69 | 45 | 16 | 62 |
| ‘Bolagh-4’ | 34 | 7.62 | 24 | 77 | 44 | 24 | 72 |
| ‘Bolagh-5’ | 35 | 7.67 | 42 | 48 | 43 | 42 | 53 |
| ‘Bolagh-6’ | 36 | 7.79 | 41 | 47 | 42 | 41 | 70 |
| ‘Bolagh-7’ | 37 | 8.35 | 9 | 67 | 41 | 9 | 55 |
| ‘Bolagh-8’ | 38 | 8.41 | 21 | 74 | 40 | 21 | 51 |
| Bolagh-9’ | 39 | 8.48 | 10 | 68 | 39 | 10 | 60 |
| ‘Bolagh-10’ | 40 | 8.56 | 8 | 28 | 38 | 8 | 53 |
| ‘Alibolaghi-1’ | 41 | 8.63 | 22 | 75 | 37 | 22 | 51 |
| ‘Alibolaghi-2’ | 42 | 8.84 | 7 | 65 | 36 | 7 | 67 |

**S1 Table.** Continued.

| Accession | Stage | Distance | Cluster1 | Cluster2 | Number of Clusters | New Cluster | Next Stage |
| --- | --- | --- | --- | --- | --- | --- | --- |
| ‘Alibolaghi-3’ | 43 | 8.97 | 5 | 63 | 35 | 5 | 66 |
| ‘Alibolaghi-4’ | 44 | 9.27 | 45 | 51 | 34 | 45 | 59 |
| ‘Alibolaghi-5’ | 45 | 9.45 | 23 | 76 | 33 | 23 | 63 |
| ‘Alibolaghi-6’ | 46 | 9.73 | 19 | 72 | 32 | 19 | 69 |
| ‘Alibolaghi-7’ | 47 | 10.04 | 6 | 64 | 31 | 6 | 72 |
| ‘Alibolaghi-8’ | 48 | 10.41 | 20 | 73 | 30 | 20 | 54 |
| ‘Alibolaghi-9’ | 49 | 10.66 | 4 | 62 | 29 | 4 | 66 |
| ‘Alibolaghi-10’ | 50 | 12.05 | 3 | 61 | 28 | 3 | 59 |
| ‘Palangdarreh-1’ | 51 | 12.15 | 21 | 22 | 27 | 21 | 58 |
| ‘Palangdarreh-2’ | 52 | 12.63 | 2 | 60 | 26 | 2 | 74 |
| ‘Palangdarreh-3’ | 53 | 14.16 | 8 | 42 | 25 | 8 | 56 |
| ‘Palangdarreh-4’ | 54 | 14.55 | 17 | 20 | 24 | 17 | 65 |
| ‘Palangdarreh-5’ | 55 | 15.23 | 9 | 18 | 23 | 9 | 60 |
| ‘Palangdarreh-6’ | 56 | 15.50 | 8 | 25 | 22 | 8 | 58 |
| ‘Palangdarreh-7’ | 57 | 15.54 | 1 | 59 | 21 | 1 | 74 |
| ‘Palangdarreh-8’ | 58 | 16.45 | 8 | 21 | 20 | 8 | 64 |
| ‘Palangdarreh-9’ | 59 | 16.82 | 3 | 45 | 19 | 3 | 63 |
| ‘Palangdarreh-10’ | 60 | 17.01 | 9 | 10 | 18 | 9 | 65 |
| ‘Savarabad-1’ | 61 | 17.37 | 44 | 46 | 17 | 44 | 76 |
| ‘Savarabad-2’ | 62 | 17.79 | 16 | 43 | 16 | 16 | 64 |
| ‘Savarabad-3’ | 63 | 18.19 | 3 | 23 | 15 | 3 | 67 |
| ‘Savarabad-4’ | 64 | 18.44 | 8 | 16 | 14 | 8 | 68 |
| ‘Savarabad-5’ | 65 | 19.47 | 9 | 17 | 13 | 9 | 68 |
| ‘Savarabad-6’ | 66 | 19.73 | 4 | 5 | 12 | 4 | 73 |
| ‘Savarabad-7’ | 67 | 20.32 | 3 | 7 | 11 | 3 | 71 |
| ‘Savarabad-8’ | 68 | 21.97 | 8 | 9 | 10 | 8 | 69 |
| ‘Savarabad-9’ | 69 | 25.15 | 8 | 19 | 9 | 8 | 70 |
| ‘Savarabad-10’ | 70 | 26.43 | 8 | 41 | 8 | 8 | 71 |
| ‘Baneh-1’ | 71 | 27.63 | 3 | 8 | 7 | 3 | 73 |
| ‘Baneh-2’ | 72 | 28.28 | 6 | 24 | 6 | 6 | 75 |
| ‘Baneh-3’ | 73 | 29.82 | 3 | 4 | 5 | 3 | 77 |
| ‘Baneh-4’ | 74 | 30.09 | 1 | 2 | 4 | 1 | 75 |
| ‘Baneh-5’ | 75 | 34.49 | 1 | 6 | 3 | 1 | 77 |
| ‘Baneh-6’ | 76 | 36.34 | 3 | 44 | 2 | 3 | 77 |
| ‘Baneh-7’ | 77 | 43.36 | 1 | 3 | 1 | 1 | -- |
| ‘Baneh-8’ | 78 | - | - | - | - | - | -- |

Distance type: Euclidean.
