# Supplementary material for: Phenotypic variations of naturally grown Iris persica L. accessions as revealed using multivariate analytical methods
Source: PLoS One. 2026 Jul 27;21(7):e0354156. doi: 10.1371/journal.pone.0354156 (PMC13405088; doi:10.1371/journal.pone.0354156)
Supplement: Attachment — Submitted filename: Response to the Editor and Reviewers.docx [file pone.0354156.s004.docx]

**General Response to the Editor and Reviewers**

**We would like to express our sincere gratitude to the Editor and Reviewers for their valuable time, effort, and constructive comments. We greatly appreciate the Editor’s careful evaluation of our manuscript, thoughtful management of the editorial process, and insightful suggestions that have significantly contributed to improving the scientific quality, clarity, and overall presentation of the work. We have carefully considered all comments and recommendations provided by the Editor and Reviewers, and the manuscript has been revised accordingly. To facilitate the review of the revised version, all modifications have been highlighted using different colors according to their source:**

**Yellow: Changes made in response to the Editor’s comments and suggestions.**

**Green: Changes made in response to Reviewer 1’s comments and suggestions.**

**Turquoise: Changes made in response to Reviewer 2’s comments and suggestions.**

**Pink: Changes made in response to Reviewer 3’s comments and suggestions.**

**We hope that the revisions satisfactorily address all concerns raised by the Editor and Reviewers, and we sincerely thank them again for their constructive feedback, which has substantially strengthened the manuscript.**

**Regards,**

**Yazgan Tunç, Ph. D., Corresponding author**

**For Editor:**

**Editor, Comment 1:** The Introduction should clearly define the research gap, hypothesis, originality, and rationale of the study.

**Response:** Thank you for this constructive comment. The Introduction has been revised to more clearly identify the existing research gap, state the study rationale, and highlight the originality of the work. We also strengthened the objectives of the study and clarified the underlying hypothesis regarding phenotypic variation among natural populations of Iris persica.

**Editor, Comment 2: Materials and Methods should include a clearer description of the experimental design, replication strategy, growth stage of evaluation, and supporting references.**

**Response:** Thank you for this suggestion. The Materials and Methods section was revised and expanded to clarify the sampling procedure, evaluation stage, morphological assessment methods, statistical analyses, and supporting references, thereby improving the transparency and reproducibility of the study.

**Editor, Comment 3:** The manuscript requires improvements in statistical reporting, including the inclusion of ANOVA tables and mean comparisons.

**Response:** We sincerely thank the Editor for this valuable comment. We carefully reconsidered the statistical framework of the study and revised the manuscript accordingly.

The primary objective of the present research was to characterize phenotypic diversity and explore patterns of morphological variation among naturally occurring Iris persica accessions using multivariate analytical approaches rather than to compare treatment means under an experimental design. Since each accession represented a distinct wild-collected individual and the study was conducted as a germplasm characterization survey rather than a replicated experimental trial, we determined that the inclusion of ANOVA tables and mean comparison tests was not the most appropriate analytical approach for addressing the study objectives.

Therefore, we revised the statistical analysis section to focus on descriptive statistics (minimum, maximum, mean, standard deviation, and coefficient of variation), correlation analysis, principal component analysis (PCA), hierarchical cluster analysis (HCA), and multiple regression analysis (MRA), which are widely employed in morphological diversity and germplasm characterization studies. These methods provide a more suitable framework for identifying phenotypic variability, trait associations, and patterns of accession differentiation.

Accordingly, references to one-way ANOVA, assumption testing procedures, and associated significance statements were removed from both the Materials and Methods and Results sections. The revised manuscript now presents descriptive statistical parameters generated using OriginPro® 2025b (OriginLab, 2025), together with multivariate analyses that directly support the objectives of phenotypic characterization, conservation assessment, and identification of superior accessions.

In addition, to enhance transparency, reproducibility, and compliance with journal data-sharing policies, all raw data associated with this study have been deposited in the Zenodo public repository and are freely accessible through the following DOI:

[**https://doi.org/10.5281/zenodo.20809887**](https://doi.org/10.5281/zenodo.20809887)

We believe that these revisions have improved the methodological consistency of the manuscript and better aligned the statistical analyses with the overall aims of the study.

**Editor, Comment 4:**  Please upload a new copy of Figure 5 and Figure 6, as the detail is not clear.

**Response:** Thank you for this valuable suggestion. Figures 5 and 6 were replaced with higher-resolution versions (900 DPI instead of 300 DPI) to substantially improve image quality and readability. In addition, the accession labels in Figure 6 were abbreviated to enhance visual clarity and facilitate interpretation. Specifically, accession names were shortened as follows: Sorkhe (So), Shahbaz (Sh), Bolagh (Bo), Alibolaghi (Al), Palangdarreh (Pa), Savarabad (Sa), and Baneh (Ba), while the corresponding genotype numbers were retained unchanged. The abbreviations used were clearly defined in the figure caption to ensure transparency and ease of interpretation.

**For Reviewer 1:**

**Reviewer 1, Comment 1:** The type and component of the experimental design should be defined.

Response: Thank you for this valuable comment. Since the present study was based on the morphological characterization of naturally occurring wild accessions, no formal experimental design (e.g., CRD or RCBD) was applied. Each accession represented a single independently growing individual collected from natural habitats. To clarify the study design, a statement describing the observational sampling approach, the number of accessions (78), the number of collection regions (seven), and the evaluated traits (39 morphological and colorimetric characters) has been added to the Abstract.

**Reviewer 1, Comment 2:** Some lines about the genetic diversity methods should be provided.

**Response:** Thank you for this valuable suggestion. Additional information describing the main approaches used to assess genetic diversity in plant populations, including morphological, molecular, and multivariate analytical methods, has been incorporated into the Introduction section. Relevant references have also been added to support this information.

**Reviewer 1, Comment 3:** Some lines about the conservation strategies of this plant species should be added.

**Response:** We appreciate the reviewer’s suggestion. A new paragraph highlighting the importance of in situ and ex situ conservation strategies for *Iris persica* and the role of phenotypic diversity studies in germplasm conservation and sustainable utilization has been added to the Introduction.

**Reviewer 1, Comment 4:** The gap and hypothesis of the study should be defined.

**Response:** Thank you for this important comment. The knowledge gap addressed by the present study and the corresponding research hypothesis have now been clearly stated in the final part of the Introduction to better justify the objectives and significance of the work.

**Reviewer 1, Comment 5:** The originality of the work should be determined.

**Response:** We agree with the reviewer. The novelty and originality of the study have been explicitly emphasized in the Introduction by highlighting the limited available information on naturally growing *Iris persica* populations and the comprehensive multivariate evaluation performed in the present investigation.

**Reviewer 1, Comment 6:** Some references related to this work should be stated.

**Response:** Thank you for the suggestion. Several additional and relevant references have been incorporated throughout the Introduction to strengthen the scientific background and provide a broader context for the study.

**Reviewer 1, Comment 7:** All abbreviations should be mentioned in full name at the first writing.

**Response:** Thank you for this suggestion. All abbreviations used throughout the manuscript were carefully checked and are now provided in their full form at first mention, followed by the corresponding abbreviation in parentheses. The manuscript has been revised accordingly.

**Reviewer 1, Comment 8:** All methodology used should be supported by references.

**Response:** We appreciate the reviewer’s comment. Appropriate references have been added throughout the Materials and Methods section to support all methodological procedures, including morphological measurements, color assessment, statistical analyses, and multivariate evaluation methods.

**Reviewer 1, Comment 9:** The growth stage of each measurement should be defined.

**Response:** Thank you for this important observation. The developmental stage at which all measurements were performed has now been clearly specified in the Materials and Methods section. All morphological evaluations were conducted during the flowering stage to ensure consistency among accessions.

**Reviewer 1, Comment 10:** The number of replicates for each measurement should be specified.

**Response:** Thank you for the valuable comment. Additional clarification has been provided in the Materials and Methods section. Each accession was treated as a single biological replicate because the study was conducted on naturally occurring wild populations, and all morphological measurements were recorded during the flowering stage.

**Reviewer 1, Comment 11:** The status of significance should be indicated at the beginning of text for each measurement.

**Response:** Thank you for this valuable comment. We carefully reconsidered the statistical framework of the study. Because the present work was designed as a phenotypic characterization survey of naturally occurring wild accessions rather than a replicated experimental trial, significance testing for individual traits was not considered the most appropriate analytical approach. Therefore, references to ANOVA-based significance statements were removed from the revised manuscript, and greater emphasis was placed on descriptive statistics and multivariate analyses, which more directly address the objectives of phenotypic diversity assessment and accession differentiation.

**Reviewer 1, Comment 12:** ANOVA table should be provided.

**Response:** Thank you for this suggestion. Following a careful re-evaluation of the statistical approach, we concluded that the inclusion of an ANOVA table was not appropriate for the objectives and structure of the present study. Since each accession represented a unique wild-collected individual and the study was conducted as a germplasm characterization survey rather than a replicated experimental design, the revised manuscript focuses on descriptive statistics, correlation analysis, principal component analysis, hierarchical cluster analysis, and multiple regression analysis. These approaches are widely used and considered more suitable for morphological diversity and germplasm characterization studies.

**Reviewer 1, Comment 13:** A table about the means comparison should be stated.

**Response:** We appreciate the reviewer’s suggestion. Mean comparison procedures are generally associated with ANOVA-based experimental analyses involving replicated treatments. Because the present study was based on the characterization of naturally occurring wild accessions rather than treatment comparisons, mean comparison tests were not considered appropriate. Accordingly, the revised manuscript emphasizes descriptive and multivariate statistical approaches that better reflect the nature of the dataset and the objectives of the study.

**Reviewer 1, Comment 14:** The captions should reflect the content of the figure and table, so the captions should be improved.

**Response:** Thank you for this constructive suggestion. The captions of all tables and figures were carefully revised and expanded to provide more informative descriptions of their contents, thereby improving clarity, readability, and interpretation of the presented results.

**Reviewer 1, Comment 15:** In Figure 6, the abbreviations V1, V2… etc should be used instead of full name of the accession because the figure is too compacted.

**Response:** Thank you for this valuable comment. We agree that the original figure was visually crowded. Instead of replacing accession names with generic labels (V1, V2, etc.), which would reduce biological interpretability, accession names were abbreviated according to their collection sites (So = Sorkhe, Sh = Shahbaz, Bo = Bolagh, Al = Alibolaghi, Pa = Palangdarreh, Sa = Savarabad, and Ba = Baneh), while the original genotype numbers were retained. These abbreviations were explicitly defined in the figure caption. This revision substantially improved figure readability while preserving accession identity and traceability. Ayrıca, the figure was replaced with a higher-resolution version to further enhance visual clarity.

**Reviewer 1, Comment 16:** Additional works about this subject should be stated.

**Response:** Thank you for this valuable suggestion. The Conclusions section has been revised to further emphasize future research directions. Specifically, we highlighted the need for multi-year evaluations, molecular marker-based studies, assessment of genotype × environment interactions, and investigations into the ornamental performance and propagation potential of the superior accessions identified in the present study. These additions provide a clearer framework for the future conservation, breeding, and utilization of *Iris persica* germplasm.

**For Reviewer 2:**

**Reviewer 2, Comment 1:** The manuscript is well-written with a good interest to the readers. You can also refer to and cite the following literature:

Akin et al. (2022)

Beyhan et al. (2016)

**Response:** We sincerely thank the reviewer for this valuable suggestion. Both recommended references were carefully reviewed and found highly relevant to the multivariate analytical approaches employed in the present study. Accordingly, the suggested articles have been incorporated into the revised manuscript and cited in the Introduction section to strengthen the methodological background and support the application of multivariate statistical techniques for the evaluation of phenotypic diversity and trait relationships. The corresponding references have also been added to the References section.

**For Reviewer 3:**

**Reviewer 3, Comment 1:** Clarify sampling strategy and population representation. The authors state that accessions were sampled at least 200 m apart to avoid collecting clones. However, it would be useful to provide a stronger justification or supporting reference demonstrating that this distance is sufficient to minimize the probability of sampling genetically identical individuals in Iris persica populations.

**Response:** Thank you for this valuable suggestion. We have revised the Materials and Methods section to provide a clearer justification for the sampling strategy. Additional text has been included explaining that a minimum distance of 200 m between sampled plants was adopted to reduce the likelihood of collecting genetically identical individuals and to maximize the representation of phenotypic variation within and among natural populations. Furthermore, we have added supporting references from previous studies on wild *Iris* species that successfully employed the same sampling approach (Khaleghi et al., 2025; Khaleghi et al., 2026).

**Reviewer 3, Comment 2:** Provide references for trait descriptors. Morphological descriptors used in the study should be linked to previously published Iris characterization protocols, UPOV descriptors, or other recognized taxonomic standards where applicable.

**Response:** Thank you for this valuable suggestion. To improve the transparency and reproducibility of the morphological evaluations, we have revised the Materials and Methods section and added references supporting the descriptor system used in this study. Specifically, we clarified that the morphological trait descriptors and scoring criteria were adopted from previously published characterization studies on wild *Iris* germplasm (Khaleghi et al., 2025; Khaleghi et al., 2026), which employed comparable descriptor sets and assessment procedures.

**Reviewer 3, Comment 3:** Clarify assumptions of ANOVA. Although normality and homogeneity tests are mentioned, the manuscript would benefit from reporting the overall outcomes of these diagnostic tests, either briefly in the text or in supplementary materials.

**Response:** Thank you for this valuable comment. We carefully reconsidered the statistical framework of the study. Because the present work was designed as a phenotypic characterization survey of naturally occurring wild accessions rather than a replicated experimental trial, significance testing based on ANOVA assumptions was not considered the most appropriate analytical approach. Therefore, references to ANOVA assumption testing procedures, including normality and homogeneity diagnostics, were removed from the revised manuscript. The statistical evaluation now focuses on descriptive statistics, correlation analysis, principal component analysis (PCA), hierarchical cluster analysis (HCA), and multiple regression analysis (MRA), which are more suitable for assessing phenotypic diversity, trait relationships, and accession differentiation in germplasm characterization studies.

**Reviewer 3, Comment 4:** Report multicollinearity diagnostics in regression analyses. Because many floral traits are highly correlated, variance inflation factors (VIFs) or tolerance values should be reported to demonstrate that multicollinearity did not substantially affect the regression models.

**Response:** Thank you for this valuable suggestion. To address this concern, we calculated and incorporated multicollinearity diagnostics for all multiple regression models. Specifically, Variance Inflation Factor (VIF) and tolerance values were added to the regression table, allowing readers to evaluate the degree of collinearity among predictor variables and assess the robustness of the regression analyses.

**Reviewer 3, Comment 5:** Provide model performance statistics. For the multiple regression analyses, it would be useful to report adjusted R² values, model significance statistics, and prediction errors to allow readers to better evaluate model performance.

**Response:** Thank you for this helpful suggestion. We agree that additional model performance statistics improve the interpretation and transparency of regression analyses. Accordingly, the regression table has been revised to include adjusted R² values, model significance statistics (F-values), and prediction error estimates (standard error of estimate, SEE). These additions provide a more comprehensive assessment of model performance and facilitate the evaluation of the predictive strength and reliability of the regression models.

**Reviewer 3, Comment 6:** Consider including a supplementary accession dataset. Providing a supplementary table containing the complete trait values for each accession would increase transparency and facilitate future comparative studies, meta-analyses, and breeding applications.

**Response:** Thank you for this valuable suggestion. We agree that providing access to the complete accession dataset improves transparency, reproducibility, and the long-term utility of the study. Accordingly, all raw morphological data used in the present investigation have been deposited in the Zenodo public repository and are freely accessible through the DOI provided in the revised manuscript. This dataset contains the complete trait values for all evaluated accessions and can be used for future comparative studies, meta-analyses, and breeding applications.

**‘Availability of data and materials:**

**The datasets generated and analyzed during the current study are publicly available in the Zenodo repository at** [**https://doi.org/10.5281/zenodo.20809887**](https://doi.org/10.5281/zenodo.20809887)**.’**

**Reviewer 3, Comment 7:** Improve consistency of terminology and formatting. Minor inconsistencies were observed in the presentation of trait names, capitalization of qualitative categories, and species nomenclature. A careful editorial review is recommended to ensure uniform terminology throughout the manuscript.

**Response:** Thank you for this helpful observation. We carefully reviewed the entire manuscript and implemented a comprehensive editorial revision to improve consistency in terminology and formatting. Trait names, capitalization of qualitative categories, species nomenclature, table contents, figure captions, and formatting styles were thoroughly checked and standardized throughout the manuscript. In addition, the manuscript underwent a comprehensive language and editorial review to improve clarity, readability, and consistency of presentation. An acknowledgment statement regarding the use of ChatGPT as a language-support tool during manuscript preparation and revision has also been added in accordance with journal recommendations. These revisions have enhanced the overall quality, consistency, and readability of the manuscript.

**Reviewer 3, Comment 8:**

Overall Recommendation

The manuscript presents a valuable morphological assessment of wild *Iris persica* germplasm and contributes useful information for ornamental breeding and conservation. The issues identified above are relatively minor and can be addressed without substantial additional experimentation. Therefore, I recommend Minor Revision.

**Response:** We sincerely thank the reviewer for the positive evaluation of our work and for recognizing its potential contribution to ornamental breeding and germplasm conservation. We appreciate the constructive comments and suggestions provided. All concerns have been carefully addressed in the revised manuscript, which we believe has been substantially improved as a result.
