# Supplementary material for: Phenotypic variations of naturally grown Iris persica L. accessions as revealed using multivariate analytical methods
Source: PLoS One. 2026 Jul 27;21(7):e0354156. doi: 10.1371/journal.pone.0354156 (PMC13405088; doi:10.1371/journal.pone.0354156)
Supplement: Peer review history

# Peer review: Decision Letter 0

**DOI:** 10.1371/journal.pone.0354156.r001

**Authors:** Faham Khamesipour

19 Jun 2026

PONE-D-26-06249

Phenotypic variations of naturally grown Iris persica L. accessions as revealed using multivariate analytical methods

PLOS One

Dear Dr. Khadivi,

Thank you for submitting your manuscript to PLOS ONE. After careful consideration, we feel that it has merit but does not fully meet PLOS ONE’s publication criteria as it currently stands. Therefore, we invite you to submit a revised version of the manuscript that addresses the points raised during the review process.

Please submit your revised manuscript by Aug 03 2026 11:59PM. If you will need more time than this to complete your revisions, please reply to this message or contact the journal office at plosone@plos.org. When you're ready to submit your revision, log on to https://www.editorialmanager.com/pone/ and select the 'Submissions Needing Revision' folder to locate your manuscript file.

Please include the following items when submitting your revised manuscript:

- A letter that responds to each point raised by the academic editor and reviewer(s). You should upload this letter as a separate file labeled 'Response to Reviewers'.
- A marked-up copy of your manuscript that highlights changes made to the original version. You should upload this as a separate file labeled 'Revised Manuscript with Track Changes'.
- An unmarked version of your revised paper without tracked changes. You should upload this as a separate file labeled 'Manuscript'.

If you would like to make changes to your financial disclosure, please include your updated statement in your cover letter. Guidelines for resubmitting your figure files are available below the reviewer comments at the end of this letter.

If applicable, we recommend that you deposit your laboratory protocols in protocols.io to enhance the reproducibility of your results. Protocols.io assigns your protocol its own identifier (DOI) so that it can be cited independently in the future. For instructions see: https://journals.plos.org/plosone/s/submission-guidelines#loc-laboratory-protocols. Additionally, PLOS ONE offers an option for publishing peer-reviewed Lab Protocol articles, which describe protocols hosted on protocols.io. Read more information on sharing protocols at https://plos.org/protocols?utm_medium=editorial-email&utm_source=authorletters&utm_campaign=protocols.

As the corresponding author, your ORCID iD is verified in the submission system and will appear in the published article. PLOS supports the use of ORCID, and we encourage all coauthors to register for an ORCID iD and use it as well. Please encourage your coauthors to verify their ORCID iD within the submission system before final acceptance, as unverified ORCID iDs will not appear in the published article. *Only*  the individual author can complete the verification step; PLOS staff *cannot*  verify ORCID iDs on behalf of authors.

We look forward to receiving your revised manuscript.

Kind regards,

Faham Khamesipour, Ph.D.

Academic Editor

PLOS One

**Journal Requirements:**

1. When submitting your revision, we need you to address these additional requirements.

Please ensure that your manuscript meets PLOS ONE's style requirements, including those for file naming. The PLOS ONE style templates can be found at

https://journals.plos.org/plosone/s/file?id=wjVg/PLOSOne_formatting_sample_main_body.pdf and

https://journals.plos.org/plosone/s/file?id=ba62/PLOSOne_formatting_sample_title_authors_affiliations.pdf

2. Please include a complete copy of PLOS’ questionnaire on inclusivity in global research in your revised manuscript. Our policy for research in this area aims to improve transparency in the reporting of research performed outside of researchers’ own country or community. The policy applies to researchers who have travelled to a different country to conduct research, research with Indigenous populations or their lands, and research on cultural artefacts. The questionnaire can also be requested at the journal’s discretion for any other submissions, even if these conditions are not met.  Please find more information on the policy and a link to download a blank copy of the questionnaire here: https://journals.plos.org/plosone/s/best-practices-in-research-reporting. Please upload a completed version of your questionnaire as Supporting Information when you resubmit your manuscript.

3. In your Methods section, please provide additional information regarding the permits you obtained for the work. Please ensure you have included the full name of the authority that approved the field site access and, if no permits were required, a brief statement explaining why.

4. We note that Figure 1 in your submission contain map images which may be copyrighted. All PLOS content is published under the Creative Commons Attribution License (CC BY 4.0), which means that the manuscript, images, and Supporting Information files will be freely available online, and any third party is permitted to access, download, copy, distribute, and use these materials in any way, even commercially, with proper attribution. For these reasons, we cannot publish previously copyrighted maps or satellite images created using proprietary data, such as Google software (Google Maps, Street View, and Earth). For more information, see our copyright guidelines: http://journals.plos.org/plosone/s/licenses-and-copyright.

We require you to either present written permission from the copyright holder to publish these figures specifically under the CC BY 4.0 license, or remove the figures from your submission:

a. You may seek permission from the original copyright holder of Figure 1 to publish the content specifically under the CC BY 4.0 license.

We recommend that you contact the original copyright holder with the Content Permission Form (http://journals.plos.org/plosone/s/file?id=7c09/content-permission-form.pdf) and the following text:

“I request permission for the open-access journal PLOS ONE to publish XXX under the Creative Commons Attribution License (CCAL) CC BY 4.0 (http://creativecommons.org/licenses/by/4.0/). Please be aware that this license allows unrestricted use and distribution, even commercially, by third parties. Please reply and provide explicit written permission to publish XXX under a CC BY license and complete the attached form.”

Please upload the completed Content Permission Form or other proof of granted permissions as an "Other" file with your submission.

In the figure caption of the copyrighted figure, please include the following text: “Reprinted from [ref] under a CC BY license, with permission from [name of publisher], original copyright [original copyright year].”

b. If you are unable to obtain permission from the original copyright holder to publish these figures under the CC BY 4.0 license or if the copyright holder’s requirements are incompatible with the CC BY 4.0 license, please either i) remove the figure or ii) supply a replacement figure that complies with the CC BY 4.0 license. Please check copyright information on all replacement figures and update the figure caption with source information. If applicable, please specify in the figure caption text when a figure is similar but not identical to the original image and is therefore for illustrative purposes only.

The following resources for replacing copyrighted map figures may be helpful:

USGS National Map Viewer (public domain): http://viewer.nationalmap.gov/viewer/

The Gateway to Astronaut Photography of Earth (public domain): http://eol.jsc.nasa.gov/sseop/clickmap/

Maps at the CIA (public domain): https://www.cia.gov/library/publications/the-world-factbook/index.html and https://www.cia.gov/library/publications/cia-maps-publications/index.html

NASA Earth Observatory (public domain): http://earthobservatory.nasa.gov/

Landsat: http://landsat.visibleearth.nasa.gov/

USGS EROS (Earth Resources Observatory and Science (EROS) Center) (public domain): http://eros.usgs.gov/#

Natural Earth (public domain): http://www.naturalearthdata.com/

5. We note that Figures 2, 3 and 4 in your submission contain copyrighted images. All PLOS content is published under the Creative Commons Attribution License (CC BY 4.0), which means that the manuscript, images, and Supporting Information files will be freely available online, and any third party is permitted to access, download, copy, distribute, and use these materials in any way, even commercially, with proper attribution. For more information, see our copyright guidelines: http://journals.plos.org/plosone/s/licenses-and-copyright.

We require you to either present written permission from the copyright holder to publish these figures specifically under the CC BY 4.0 license, or remove the figures from your submission:

a. You may seek permission from the original copyright holder of Figures 2, 3 and 4 to publish the content specifically under the CC BY 4.0 license.

We recommend that you contact the original copyright holder with the Content Permission Form (http://journals.plos.org/plosone/s/file?id=7c09/content-permission-form.pdf) and the following text:

“I request permission for the open-access journal PLOS ONE to publish XXX under the Creative Commons Attribution License (CCAL) CC BY 4.0 (http://creativecommons.org/licenses/by/4.0/). Please be aware that this license allows unrestricted use and distribution, even commercially, by third parties. Please reply and provide explicit written permission to publish XXX under a CC BY license and complete the attached form.”

Please upload the completed Content Permission Form or other proof of granted permissions as an "Other" file with your submission.

In the figure caption of the copyrighted figure, please include the following text: “Reprinted from [ref] under a CC BY license, with permission from [name of publisher], original copyright [original copyright year].”

b. If you are unable to obtain permission from the original copyright holder to publish these figures under the CC BY 4.0 license or if the copyright holder’s requirements are incompatible with the CC BY 4.0 license, please either i) remove the figure or ii) supply a replacement figure that complies with the CC BY 4.0 license. Please check copyright information on all replacement figures and update the figure caption with source information. If applicable, please specify in the figure caption text when a figure is similar but not identical to the original image and is therefore for illustrative purposes only.

6. Please upload a new copy of Figures 5 and 6, as the detail is not clear. Please follow the link for more information:  https://journals.plos.org/plosone/s/figures

7. If the reviewer comments include a recommendation to cite specific previously published works, please review and evaluate these publications to determine whether they are relevant and should be cited. There is no requirement to cite these works unless the editor has indicated otherwise.

**Additional Editor Comments:**

After careful evaluation of the manuscript entitled “Phenotypic variations of Iris persica L. as revealed using morphological and phenological characterizations” and considering the reports from the reviewers, the manuscript addresses an interesting and relevant topic related to the phenotypic diversity and potential conservation of Iris persica germplasm.

While one reviewer finds the manuscript generally well-structured and suitable for publication with only minor revisions, the other reviewer raises several important concerns regarding methodological clarity, statistical reporting, interpretation of results, and the overall novelty of the study. In particular, improvements are needed in the introduction (clear definition of research gap, hypothesis, originality, and supporting literature), materials and methods (full description of experimental design, replication, growth stages, and proper referencing), and results and discussion (inclusion of ANOVA tables, mean comparisons, clearer figure/table captions, and stronger interpretation of relationships among traits). The discussion section, in particular, requires substantial improvement, especially in interpreting results in the context of conservation strategies for the species.

In addition, clarification and standardization of abbreviations, as well as improved presentation of figures (e.g., accession coding in Figure 6), are necessary to enhance readability and scientific rigor.

Although the study has potential and the subject is of interest to the readership, the extent of the required revisions goes beyond minor corrections.

The manuscript requires major revision before it can be reconsidered for publication. The authors are encouraged to carefully address all reviewer comments in a detailed and point-by-point response letter. If properly revised, the manuscript may be suitable for publication in a future round of review.

[Note: HTML markup is below. Please do not edit.]

Reviewers' comments:

Reviewer's Responses to Questions

**Comments to the Author**

1. Is the manuscript technically sound, and do the data support the conclusions?

The manuscript must describe a technically sound piece of scientific research with data that supports the conclusions. Experiments must have been conducted rigorously, with appropriate controls, replication, and sample sizes. The conclusions must be drawn appropriately based on the data presented.

Reviewer #1: No

Reviewer #2: Yes

Reviewer #3: Yes

**********

2. Has the statistical analysis been performed appropriately and rigorously?

Reviewer #1: No

Reviewer #2: Yes

Reviewer #3: Yes

**********

3. Have the authors made all data underlying the findings in their manuscript fully available?

The PLOS Data policy requires authors to make all data underlying the findings described in their manuscript fully available without restriction, with rare exception (please refer to the Data Availability Statement in the manuscript PDF file). The data should be provided as part of the manuscript or its supporting information, or deposited to a public repository. For example, in addition to summary statistics, the data points behind means, medians and variance measures should be available. If there are restrictions on publicly sharing data—e.g. participant privacy or use of data from a third party—those must be specified.

Reviewer #1: No

Reviewer #2: Yes

Reviewer #3: Yes

**********

4. Is the manuscript presented in an intelligible fashion and written in standard English?

PLOS ONE does not copyedit accepted manuscripts, so the language in submitted articles must be clear, correct, and unambiguous. Any typographical or grammatical errors should be corrected at revision, so please note any specific errors here.

Reviewer #1: No

Reviewer #2: Yes

Reviewer #3: Yes

**********

5. Review Comments to the Author

Please use the space provided to explain your answers to the questions above. You may also include additional comments for the author, including concerns about dual publication, research ethics, or publication ethics. (Please upload your review as an attachment if it exceeds 20,000 characters)

**Reviewer #1:**  The authors investigated the phenotypic variations of naturally grown Iris persica L. accessions as revealed using multivariate analytical methods. There is not too much novalty in this study. The following points should be fixed.

Abstract

• The type and component of the experimental design should be defined

Introduction

• Some lines about the genetic diversit methods should be provided

• Some lines about the conservation strategies of this plant species should be added

• The gap and hypothesis of the study should defined

• The orginality of the work should be determined

• Some references related to this work should be stated

Materials and Methods

• All abbreviations should be mentioned in full name at the first writing

• All methodology used should be supported by references

• The growth stage of each measurement should be defined

• The number of replicates for each measurement should be specified

Results and discussion

• The status of significance should be indicated at the beginning of text for each measurement

• ANOVA table should be provided

• A table about the means comparison should be stated

• The captions should reflect the content of the figure and table, so the captions should be improved

• In Figure 6, the abbreviations V1, V2… etc should be used instead of full name of the accession because the figure is too compacted

• Discussion: this discussion is poor and needs improvement. The authors should interpret the relationship between all studied characters and all treatments. The authors should discuss the conservation strategies for this plant species

Conclusion

Additional works about this subject should be stated

**Reviewer #2:**  The manuscript is well-written with a good interest to the readers. You can also refer to and cite the following literature:

Akin, M., Eyduran, S. P., Gazioglu Sensoy, R. I., & Eyduran, E. (2022). Defining associations between berry features of wild red currant accessions utilizing various statistical methods. Erwerbs-Obstbau, 64(3), 377-386.

Beyhan, O., Eyduran, E., Akin, M., Ercisli, S., Gecer, K. M., & Karahan, E. A. (2016). Identification of the relationship between some characteristics of native walnut genotypes peculiar to Darende district of Malatya province: Use of factor analysis scores in multiple linear regression. Genetika, 48(3), 923-932.

**Reviewer #3:**  larify sampling strategy and population representation

The authors state that accessions were sampled at least 200 m apart to avoid collecting clones. However, it would be useful to provide a stronger justification or supporting reference demonstrating that this distance is sufficient to minimize the probability of sampling genetically identical individuals in Iris persica populations.

Provide references for trait descriptors

Morphological descriptors used in the study should be linked to previously published Iris characterization protocols, UPOV descriptors, or other recognized taxonomic standards where applicable.

Clarify assumptions of ANOVA

Although normality and homogeneity tests are mentioned, the manuscript would benefit from reporting the overall outcomes of these diagnostic tests, either briefly in the text or in supplementary materials.

Report multicollinearity diagnostics in regression analyses

Because many floral traits are highly correlated, variance inflation factors (VIFs) or tolerance values should be reported to demonstrate that multicollinearity did not substantially affect the regression models.

Provide model performance statistics

For the multiple regression analyses, it would be useful to report adjusted R² values, model significance statistics, and prediction errors to allow readers to better evaluate model performance.

Consider including a supplementary accession dataset

Providing a supplementary table containing the complete trait values for each accession would increase transparency and facilitate future comparative studies, meta-analyses, and breeding applications.

Improve consistency of terminology and formatting

Minor inconsistencies were observed in the presentation of trait names, capitalization of qualitative categories, and species nomenclature. A careful editorial review is recommended to ensure uniform terminology throughout the manuscript.

Overall Recommendation

The manuscript presents a valuable morphological assessment of wild Iris persica germplasm and contributes useful information for ornamental breeding and conservation. The issues identified above are relatively minor and can be addressed without substantial additional experimentation. Therefore, I recommend Minor Revision.

**********

6. PLOS authors have the option to publish the peer review history of their article (what does this mean?). If published, this will include your full peer review and any attached files.

If you choose “no”, your identity will remain anonymous but your review may still be made public.

**Do you want your identity to be public for this peer review?**  For information about this choice, including consent withdrawal, please see our Privacy Policy.

Reviewer #1: **Yes:**  Nawroz Tahir

Reviewer #2: No

Reviewer #3: No

**********

[NOTE: If reviewer comments were submitted as an attachment file, they will be attached to this email and accessible via the submission site. Please log into your account, locate the manuscript record, and check for the action link "View Attachments". If this link does not appear, there are no attachment files.]

To ensure your figures meet our technical requirements, please review our figure guidelines: https://journals.plos.org/plosone/s/figures

You may also use PLOS’s free figure tool, NAAS, to help you prepare publication quality figures: https://journals.plos.org/plosone/s/figures#loc-tools-for-figure-preparation.

NAAS will assess whether your figures meet our technical requirements by comparing each figure against our figure specifications.

# Author comment: Author response to Decision Letter 1

**DOI:** 10.1371/journal.pone.0354156.r002

24 Jun 2026

General Response to the Editor and Reviewers

We would like to express our sincere gratitude to the Editor and Reviewers for their valuable time, effort, and constructive comments. We greatly appreciate the Editor’s careful evaluation of our manuscript, thoughtful management of the editorial process, and insightful suggestions that have significantly contributed to improving the scientific quality, clarity, and overall presentation of the work. We have carefully considered all comments and recommendations provided by the Editor and Reviewers, and the manuscript has been revised accordingly. To facilitate the review of the revised version, all modifications have been highlighted using different colors according to their source:

Yellow: Changes made in response to the Editor’s comments and suggestions.

Green: Changes made in response to Reviewer 1’s comments and suggestions.

Turquoise: Changes made in response to Reviewer 2’s comments and suggestions.

Pink: Changes made in response to Reviewer 3’s comments and suggestions.

We hope that the revisions satisfactorily address all concerns raised by the Editor and Reviewers, and we sincerely thank them again for their constructive feedback, which has substantially strengthened the manuscript.

Regards,

Yazgan Tunç, Ph. D., Corresponding author

For Editor:

Editor, Comment 1: The Introduction should clearly define the research gap, hypothesis, originality, and rationale of the study.

Response: Thank you for this constructive comment. The Introduction has been revised to more clearly identify the existing research gap, state the study rationale, and highlight the originality of the work. We also strengthened the objectives of the study and clarified the underlying hypothesis regarding phenotypic variation among natural populations of Iris persica.

Editor, Comment 2: Materials and Methods should include a clearer description of the experimental design, replication strategy, growth stage of evaluation, and supporting references.

Response: Thank you for this suggestion. The Materials and Methods section was revised and expanded to clarify the sampling procedure, evaluation stage, morphological assessment methods, statistical analyses, and supporting references, thereby improving the transparency and reproducibility of the study.

Editor, Comment 3: The manuscript requires improvements in statistical reporting, including the inclusion of ANOVA tables and mean comparisons.

Response: We sincerely thank the Editor for this valuable comment. We carefully reconsidered the statistical framework of the study and revised the manuscript accordingly.

The primary objective of the present research was to characterize phenotypic diversity and explore patterns of morphological variation among naturally occurring Iris persica accessions using multivariate analytical approaches rather than to compare treatment means under an experimental design. Since each accession represented a distinct wild-collected individual and the study was conducted as a germplasm characterization survey rather than a replicated experimental trial, we determined that the inclusion of ANOVA tables and mean comparison tests was not the most appropriate analytical approach for addressing the study objectives.

Therefore, we revised the statistical analysis section to focus on descriptive statistics (minimum, maximum, mean, standard deviation, and coefficient of variation), correlation analysis, principal component analysis (PCA), hierarchical cluster analysis (HCA), and multiple regression analysis (MRA), which are widely employed in morphological diversity and germplasm characterization studies. These methods provide a more suitable framework for identifying phenotypic variability, trait associations, and patterns of accession differentiation.

Accordingly, references to one-way ANOVA, assumption testing procedures, and associated significance statements were removed from both the Materials and Methods and Results sections. The revised manuscript now presents descriptive statistical parameters generated using OriginPro® 2025b (OriginLab, 2025), together with multivariate analyses that directly support the objectives of phenotypic characterization, conservation assessment, and identification of superior accessions.

In addition, to enhance transparency, reproducibility, and compliance with journal data-sharing policies, all raw data associated with this study have been deposited in the Zenodo public repository and are freely accessible through the following DOI:

https://doi.org/10.5281/zenodo.20809887

We believe that these revisions have improved the methodological consistency of the manuscript and better aligned the statistical analyses with the overall aims of the study.

Editor, Comment 4: Please upload a new copy of Figure 5 and Figure 6, as the detail is not clear.

Response: Thank you for this valuable suggestion. Figures 5 and 6 were replaced with higher-resolution versions (900 DPI instead of 300 DPI) to substantially improve image quality and readability. In addition, the accession labels in Figure 6 were abbreviated to enhance visual clarity and facilitate interpretation. Specifically, accession names were shortened as follows: Sorkhe (So), Shahbaz (Sh), Bolagh (Bo), Alibolaghi (Al), Palangdarreh (Pa), Savarabad (Sa), and Baneh (Ba), while the corresponding genotype numbers were retained unchanged. The abbreviations used were clearly defined in the figure caption to ensure transparency and ease of interpretation.

For Reviewer 1:

Reviewer 1, Comment 1: The type and component of the experimental design should be defined.

Response: Thank you for this valuable comment. Since the present study was based on the morphological characterization of naturally occurring wild accessions, no formal experimental design (e.g., CRD or RCBD) was applied. Each accession represented a single independently growing individual collected from natural habitats. To clarify the study design, a statement describing the observational sampling approach, the number of accessions (78), the number of collection regions (seven), and the evaluated traits (39 morphological and colorimetric characters) has been added to the Abstract.

Reviewer 1, Comment 2: Some lines about the genetic diversity methods should be provided.

Response: Thank you for this valuable suggestion. Additional information describing the main approaches used to assess genetic diversity in plant populations, including morphological, molecular, and multivariate analytical methods, has been incorporated into the Introduction section. Relevant references have also been added to support this information.

Reviewer 1, Comment 3: Some lines about the conservation strategies of this plant species should be added.

Response: We appreciate the reviewer’s suggestion. A new paragraph highlighting the importance of in situ and ex situ conservation strategies for Iris persica and the role of phenotypic diversity studies in germplasm conservation and sustainable utilization has been added to the Introduction.

Reviewer 1, Comment 4: The gap and hypothesis of the study should be defined.

Response: Thank you for this important comment. The knowledge gap addressed by the present study and the corresponding research hypothesis have now been clearly stated in the final part of the Introduction to better justify the objectives and significance of the work.

Reviewer 1, Comment 5: The originality of the work should be determined.

Response: We agree with the reviewer. The novelty and originality of the study have been explicitly emphasized in the Introduction by highlighting the limited available information on naturally growing Iris persica populations and the comprehensive multivariate evaluation performed in the present investigation.

Reviewer 1, Comment 6: Some references related to this work should be stated.

Response: Thank you for the suggestion. Several additional and relevant references have been incorporated throughout the Introduction to strengthen the scientific background and provide a broader context for the study.

Reviewer 1, Comment 7: All abbreviations should be mentioned in full name at the first writing.

Response: Thank you for this suggestion. All abbreviations used throughout the manuscript were carefully checked and are now provided in their full form at first mention, followed by the corresponding abbreviation in parentheses. The manuscript has been revised accordingly.

Reviewer 1, Comment 8: All methodology used should be supported by references.

Response: We appreciate the reviewer’s comment. Appropriate references have been added throughout the Materials and Methods section to support all methodological procedures, including morphological measurements, color assessment, statistical analyses, and multivariate evaluation methods.

Reviewer 1, Comment 9: The growth stage of each measurement should be defined.

Response: Thank you for this important observation. The developmental stage at which all measurements were performed has now been clearly specified in the Materials and Methods section. All morphological evaluations were conducted during the flowering stage to ensure consistency among accessions.

Reviewer 1, Comment 10: The number of replicates for each measurement should be specified.

Response: Thank you for the valuable comment. Additional clarification has been provided in the Materials and Methods section. Each accession was treated as a single biological replicate because the study was conducted on naturally occurring wild populations, and all morphological measurements were recorded during the flowering stage.

Reviewer 1, Comment 11: The status of significance should be indicated at the beginning of text for each measurement.

Response: Thank you for this valuable comment. We carefully reconsidered the statistical framework of the study. Because the present work was designed as a phenotypic characterization survey of naturally occurring wild accessions rather than a replicated experimental trial, significance testing for individual traits was not considered the most appropriate analytical approach. Therefore, references to ANOVA-based significance statements were removed from the revised manuscript, and greater emphasis was placed on descriptive statistics and multivariate analyses, which more directly address the objectives of phenotypic diversity assessment and accession differentiation.

Reviewer 1, Comment 12: ANOVA table should be provided.

Response: Thank you for this suggestion. Following a careful re-evaluation of the statistical approach, we concluded that the inclusion of an ANOVA table was not appropriate for the objectives and structure of the present study. Since each accession represented a unique wild-collected individual and the study was conducted as a germplasm characterization survey rather than a replicated experimental design, the revised manuscript focuses on descriptive statistics, correlation analysis, principal component analysis, hierarchical cluster analysis, and multiple regression analysis. These approaches are widely used and considered more suitable for morphological diversity and germplasm characterization studies.

Reviewer 1, Comment 13: A table about the means comparison should be stated.

Response: We appreciate the reviewer’s suggestion. Mean comparison procedures are generally associated with ANOVA-based experimental analyses involving replicated treatments. Because the present study was based on the characterization of naturally occurring wild accessions rather than treatment comparisons, mean comparison tests were not considered appropriate. Accordingly, the revised manuscript emphasizes descriptive and multivariate statistical approaches that better reflect the nature of the dataset and the objectives of the study.

Reviewer 1, Comment 14: The captions should reflect the content of the figure and table, so the captions should be improved.

Response: Thank you for this constructive suggestion. The captions of all tables and figures were carefully revised and expanded to provide more informative descriptions of their contents, thereby improving clarity, readability, and interpretation of the presented results.

Reviewer 1, Comment 15: In Figure 6, the abbreviations V1, V2… etc should be used instead of full name of the accession because the figure is too compacted.

Response: Thank you for this valuable comment. We agree that the original figure was visually crowded. Instead of replacing accession names with generic labels (V1, V2, etc.), which would reduce biological interpretability, accession names were abbreviated according to their collection sites (So = Sorkhe, Sh = Shahbaz, Bo = Bolagh, Al = Alibolaghi, Pa = Palangdarreh, Sa = Savarabad, and Ba = Baneh), while the original genotype numbers were retained. These abbreviations were explicitly defined in the figure caption. This revision substantially improved figure readability while preserving accession identity and traceability. Ayrıca, the figure was replaced with a higher-resolution version to further enhance visual clarity.

Reviewer 1, Comment 16: Additional works about this subject should be stated.

Response: Thank you for this valuable suggestion. The Conclusions section has been revised to further emphasize future research directions. Specifically, we highlighted the need for multi-year evaluations, molecular marker-based studies, assessment of genotype × environment interactions, and investigations into the ornamental performance and propagation potential of the superior accessions identified in the present study. These additions provide a clearer framework for the future conservation, breeding, and utilization of *Iris persica* germplasm.

For Reviewer 2:

Reviewer 2, Comment 1: The manuscript is well-written with a good interest to the readers. You can also refer to and cite the following literature:

Akin et al. (2022)

Beyhan et al. (2016)

Response: We sincerely thank the reviewer for this valuable suggestion. Both recommended references were carefully reviewed and found highly relevant to the multivariate analytical approaches employed in the present study. Accordingly, the suggested articles have been incorporated into the revised manuscript and cited in the Introduction section to strengthen the methodological background and support the application of multivariate statistical techniques for the evaluation of phenotypic diversity and trait relationships. The corresponding references have also been added to the References section.

For Reviewer 3:

Reviewer 3, Comment 1: Clarify sampling strategy and population representation. The authors state that accessions were sampled at least 200 m apart to avoid collecting clones. However, it would be useful to provide a stronger justification or supporting reference demonstrating that this distance is sufficient to minimize the probability of sampling genetically identical individuals in Iris persica populations.

Response: Thank you for this valuable suggestion. We have revised the Materials and Methods section to provide a clearer justification for the sampling strategy. Additional text has been included explaining that a minimum distance of 200 m between sampled plants was adopted to reduce the likelihood of collecting genetically identical individuals and to maximize the representation of phenotypic variation within and among natural populations. Furthermore, we have added supporting references from previous studies on wild *Iris* species that successfully employed the same sampling approach (Khaleghi et al., 2025; Khaleghi et al., 2026).

Reviewer 3, Comment 2: Provide references for trait descriptors. Morphological descriptors used in the study should be linked to previously published Iris characterization protocols, UPOV descriptors, or other recognized taxonomic standards where applicable.

Response: Thank you for this valuable suggestion. To improve the transparency and reproducibility of the morphological evaluations, we have revised the Materials and Methods section and added references supporting the descriptor system used in this study. Specifically, we clarified that the morphological trait descriptors and scoring criteria were adopted from previously published characterization

Attachment Submitted filename: Response to the Editor and Reviewers.docx

# Peer review: Decision Letter 1

**DOI:** 10.1371/journal.pone.0354156.r003

**Authors:** Faham Khamesipour

5 Jul 2026

Phenotypic variations of naturally grown Iris persica L. accessions as revealed using multivariate analytical methods

PONE-D-26-06249R1

Dear Dr. Khadivi,

We’re pleased to inform you that your manuscript has been judged scientifically suitable for publication and will be formally accepted for publication once it meets all outstanding technical requirements.

Within one week, you’ll receive an e-mail detailing the required amendments. When these have been addressed, you’ll receive a formal acceptance letter and your manuscript will be scheduled for publication.

An invoice will be generated when your article is formally accepted. Please note, if your institution has a publishing partnership with PLOS and your article meets the relevant criteria, all or part of your publication costs will be covered. Please make sure your user information is up-to-date by logging into Editorial Manager at Editorial Manager® and clicking the ‘Update My Information' link at the top of the page. For questions related to billing, please contact billing support.

If your institution or institutions have a press office, please notify them about your upcoming paper to help maximize its impact. If they’ll be preparing press materials, please inform our press team as soon as possible -- no later than 48 hours after receiving the formal acceptance. Your manuscript will remain under strict press embargo until 2 pm Eastern Time on the date of publication. For more information, please contact onepress@plos.org.

Kind regards,

Faham Khamesipour, Ph.D.

Academic Editor

PLOS One

Additional Editor Comments (optional):

Reviewers' comments:

Reviewer's Responses to Questions

**Comments to the Author**

1. If the authors have adequately addressed your comments raised in a previous round of review and you feel that this manuscript is now acceptable for publication, you may indicate that here to bypass the “Comments to the Author” section, enter your conflict of interest statement in the “Confidential to Editor” section, and submit your "Accept" recommendation.

Reviewer #2: All comments have been addressed

Reviewer #3: All comments have been addressed

**********

2. Is the manuscript technically sound, and do the data support the conclusions?

The manuscript must describe a technically sound piece of scientific research with data that supports the conclusions. Experiments must have been conducted rigorously, with appropriate controls, replication, and sample sizes. The conclusions must be drawn appropriately based on the data presented.

Reviewer #2: Yes

Reviewer #3: Yes

**********

3. Has the statistical analysis been performed appropriately and rigorously?

Reviewer #2: Yes

Reviewer #3: Yes

**********

4. Have the authors made all data underlying the findings in their manuscript fully available?

The PLOS Data policy requires authors to make all data underlying the findings described in their manuscript fully available without restriction, with rare exception (please refer to the Data Availability Statement in the manuscript PDF file). The data should be provided as part of the manuscript or its supporting information, or deposited to a public repository. For example, in addition to summary statistics, the data points behind means, medians and variance measures should be available. If there are restrictions on publicly sharing data—e.g. participant privacy or use of data from a third party—those must be specified.

Reviewer #2: Yes

Reviewer #3: Yes

**********

5. Is the manuscript presented in an intelligible fashion and written in standard English?

PLOS ONE does not copyedit accepted manuscripts, so the language in submitted articles must be clear, correct, and unambiguous. Any typographical or grammatical errors should be corrected at revision, so please note any specific errors here.

Reviewer #2: Yes

Reviewer #3: Yes

**********

6. Review Comments to the Author

Please use the space provided to explain your answers to the questions above. You may also include additional comments for the author, including concerns about dual publication, research ethics, or publication ethics. (Please upload your review as an attachment if it exceeds 20,000 characters)

Reviewer #2: The manuscript looks good in its current form. All comments have been addressed. The topic is interesting with a good interest to the readers.

Reviewer #3: (No Response)

**********

7. PLOS authors have the option to publish the peer review history of their article (what does this mean?). If published, this will include your full peer review and any attached files.

If you choose “no”, your identity will remain anonymous but your review may still be made public.

**Do you want your identity to be public for this peer review?**  For information about this choice, including consent withdrawal, please see our Privacy Policy.

Reviewer #2: No

Reviewer #3: No

**********

# Editor report: Acceptance letter

**DOI:** 10.1371/journal.pone.0354156.r004

**Authors:** Faham Khamesipour

PONE-D-26-06249R1

PLOS One

Dear Dr. Khadivi,

I'm pleased to inform you that your manuscript has been deemed suitable for publication in PLOS One. Congratulations! Your manuscript is now being handed over to our production team.

At this stage, our production department will prepare your paper for publication. This includes ensuring the following:

* All references, tables, and figures are properly cited

* All relevant supporting information is included in the manuscript submission,

* There are no issues that prevent the paper from being properly typeset

You will receive further instructions from the production team, including instructions on how to review your proof when it is ready. Please keep in mind that we are working through a large volume of accepted articles, so please give us a few days to review your paper and let you know the next and final steps.

Lastly, if your institution or institutions have a press office, please let them know about your upcoming paper now to help maximize its impact. If they'll be preparing press materials, please inform our press team within the next 48 hours. Your manuscript will remain under strict press embargo until 2 pm Eastern Time on the date of publication. For more information, please contact onepress@plos.org.

You will receive an invoice from PLOS for your publication fee after your manuscript has reached the completed accept phase. If you receive an email requesting payment before acceptance or for any other service, this may be a phishing scheme. Learn how to identify phishing emails and protect your accounts at https://explore.plos.org/phishing.

If we can help with anything else, please email us at customercare@plos.org.

Thank you for submitting your work to PLOS ONE and supporting open access.

Kind regards,

PLOS ONE Editorial Office Staff

on behalf of

Dr. Faham Khamesipour

Academic Editor

PLOS One
